# Efficacy and cost-effectiveness of Internet-delivered cognitive-behavior therapy for children and adolescents with body dysmorphic disorder: study protocol for a randomized controlled trial

**DOI:** 10.1186/s13063-026-09974-z

**Published:** 2026-08-31

**Authors:** Lorena Fernández de la Cruz, Anita Birovecz, Per Andrén, Karin Melin, Kristina Aspvall, Matti Cervin, Erik Andersson, Maria Silverberg-Mörse, Martina Gumpert, Frida Wickberg, Matteo Bottai, Eva Serlachius, Daniel Rautio, David Mataix-Cols

**Affiliations:** 1 Centre for Psychiatry Research, Department of Clinical Neuroscience, Karolinska Institutet, Stockholm, Sweden; 2 Stockholm Health Care Services, Region Stockholm, Stockholm, Sweden; 3 Unit for Child and Adolescent Psychiatry, Department of Clinical Sciences, Lund University, Lund, Sweden; 4 Department of Child and Adolescent Psychiatry, Skåne University Hospital, Lund, Sweden; 5 Gillberg Neuropsychiatry Centre, Institute of Neuroscience and Physiology, Sahlgrenska Academy, University of Gothenburg, Gothenburg, Sweden; 6 Department of Pediatric Neurology and Psychiatry, Sahlgrenska University Hospital, Region Västra Götaland, Gothenburg, Sweden; 7 Division of Psychology, Department of Clinical Neuroscience, Karolinska Institutet, Stockholm, Sweden; 8 Unit of Biostatistics, Institute of Environmental Medicine, Karolinska Institutet, Stockholm, Sweden; 9 Research Department of Clinical, Educational, and Health Psychology, University College London, London, UK

**Keywords:** Body dysmorphic disorder, Dysmorphophobia, Cognitive-behavior therapy, Exposure with response prevention, Relaxation training, Internet-delivered interventions

## Abstract

**Background:**

Body dysmorphic disorder (BDD) is a prevalent and impairing mental disorder that typically onsets in adolescence. Cognitive-behavior therapy (CBT) may be effective for adolescent BDD, although the supporting evidence is currently limited. CBT for BDD is a highly specialized treatment, creating a considerable gap in access to care for young people. Therapist-guided Internet-delivered CBT (ICBT) may help bridge this gap. The primary aim of this study is to determine the efficacy of a therapist-guided ICBT program for children and adolescents with BDD versus an active comparator. Secondary aims are to examine the 6-month durability of the treatment effects and to evaluate its relative cost-effectiveness from multiple perspectives.

**Methods:**

This is a 3-site superiority randomized controlled trial including 154 young people (12–17 years) with BDD recruited throughout Sweden. Participants are randomized 1:1 to 12 weekly modules of either therapist-supported ICBT primarily based on exposure with response prevention or an active comparator consisting of therapist-supported Internet-delivered relaxation training. Data will be collected at baseline, mid-treatment, post-treatment, and 1 month (primary endpoint), 3 months, and 6 months post-treatment. The primary outcome is BDD symptom severity measured with the Yale-Brown Obsessive-Compulsive Scale Modified for Body Dysmorphic Disorder, Adolescent version. All study personnel who can be blinded to study aims/hypotheses and group allocation will be blinded. Assessors conducting post-treatment and follow-up assessments will be external to the research team and blinded to study aims/hypotheses and group allocation at all assessment points. Analyses will be conducted according to the intention-to-treat principle and will follow a pre-specified statistical and health economic analysis plan.

**Discussion:**

Participant recruitment started on 22 February 2024 and is currently ongoing. Data analysis for the primary aim will commence after the last participant reaches the primary endpoint.

**Trial registration:**

ClinicalTrials.gov NCT06262412. Registered on 16 February 2024, https://clinicaltrials.gov/study/NCT06262412.

**Supplementary Information:**

The online version contains supplementary material available at https://doi.org/10.1186/s13063-026-09974-z.

## Background

Body dysmorphic disorder (BDD) is a mental disorder characterized by intense preoccupation with perceived defects in physical appearance, leading to time-consuming repetitive behaviors and mental acts (e.g., mirror checking, camouflaging, comparing), as well as avoidance [1]. The mean age of onset of BDD is between 12 and 16 years [2, 3]. The point prevalence of BDD in youth is 1%, with the disorder being significantly more common among adolescents than in children, and more common among girls than in boys [4]. Nonetheless, the disorder often goes undetected in adolescents due to BDD symptoms often being interpreted as normal developmental concerns, lack of awareness of the disorder, and scarcity of trained clinicians who can diagnose the condition [1, 5]. Additionally, approximately half of adolescents with BDD have been shown to exhibit poor or absent insight [3, 5], which further complicates detection, as they may struggle to describe their symptoms or seek psychiatric care. BDD in adolescence is associated with marked functional impairment, social withdrawal, reduced academic performance and school disengagement, high rates of psychiatric comorbidity, and elevated risk of suicidality [3–8]. Together, these factors highlight the urgent need for early symptom detection and improved access to treatment.

Cognitive-behavior therapy (CBT) including exposure with response prevention (ERP) is the recommended treatment for BDD in clinical guidelines [9–11]. The evidence base stems from a relatively small number of randomized controlled trials (RCTs) in adults [12, 13], while studies in youth are sparse. In one small pilot RCT in children and adolescents (*N* = 30), conducted in a specialist clinic in England, participants in the CBT condition experienced a significant reduction of BDD symptoms, compared with a control psychoeducation condition, both immediately after CBT and 2 months after treatment [5]. The gains were maintained up to 12 months after the end of the intervention in a naturalistic follow-up [14]. The treatment manual used in this study was further developed and evaluated with excellent results by our research team in a naturalistic study of 140 young people with BDD treated at specialist clinics in Stockholm, Sweden and London, England [15]. This study showed that CBT delivered in regular care by specialist teams results in statistically significant reductions in BDD symptoms both at post-treatment and up to 1 year after the end of the intervention. Another RCT in 15-to-21-year-olds with BDD in Germany showed that participants randomized to an Internet-delivered CBT (ICBT) program consisting of 12 interactive self-help sessions, called ImaginYouth (*n* = 29), had a significantly larger BDD symptom reduction than those randomized to a supportive online therapy including psychoeducational information and low-threshold counseling (*n* = 16) [16]. Despite the encouraging results from these and a handful of other smaller, open studies in pediatric BDD [17, 18], the evidence supporting CBT for BDD in youth is scarce. Moreover, it is currently unclear whether CBT with ERP outperforms other psychological interventions that do not require such a high level of expertise. For example, a US-based study of 50 adults with BDD comparing an Internet-based interpretation bias modification training protocol to progressive muscle relaxation found that both treatments yielded significant reductions in BDD symptoms, with no between-group differences [19]. The field would greatly benefit from additional RCTs, particularly in young people, addressing the question of treatment specificity.

Additionally, CBT for BDD is a highly specialized treatment, often difficult to access due to lack of trained professionals and geographical barriers. A possible solution is to deliver a low-intensity online version of the treatment with minimal remote support from a clinician, similar to a therapist-guided self-help intervention [20]. This approach has the potential to be used in a stepped-care, scalable model of healthcare, making better use of the existing clinical resources [21, 22]. ICBT has a strong evidence base for a wide range of disorders and has been shown to be cost-effective, needing less therapist time per patient than face-to-face CBT [23–25]. A small body of literature also supports the efficacy of ICBT for adults with BDD. A Swedish program, called BDD-NET, was successfully evaluated in an RCT (*N* = 94) where ICBT was compared to supportive therapy [26], with response rates in line with face-to-face CBT and sustained treatment effects up to two years post-treatment [27]. In the USA, an app-based intervention was also successfully evaluated in an RCT for adults with BDD (*N* = 80) [28]. We have recently conducted an uncontrolled feasibility trial (*N* = 20) of an ICBT program for 12-to-17-year-olds with BDD showing that the intervention is feasible, acceptable, and safe [29]. Preliminary efficacy results showed statistically significant BDD symptom severity reductions (*d* = 2.94), with treatment gains that continued to accrue up to the 12-month follow-up [29].

The primary aim of this RCT is to evaluate the clinical efficacy of a previously piloted [29], therapist-guided ICBT program for adolescent BDD versus an active comparator consisting of Internet-delivered relaxation training (IRT) for BDD. Secondary aims include examining the 6-month durability of the treatment effects in a naturalistic follow-up and conducting a health economic evaluation of ICBT for BDD. We hypothesize that ICBT will be superior to IRT in reducing BDD symptom severity, that the effects of ICBT will be durable, and that ICBT will be more cost-effective than IRT.

## Methods

The reporting of this protocol follows the 2025 Standard Protocol Items: Recommendations for Interventional Trials (SPIRIT) statement [30]. The SPIRIT checklist can be found in Supplementary Table 1.

### Study design and setting

**Fig. 1:**
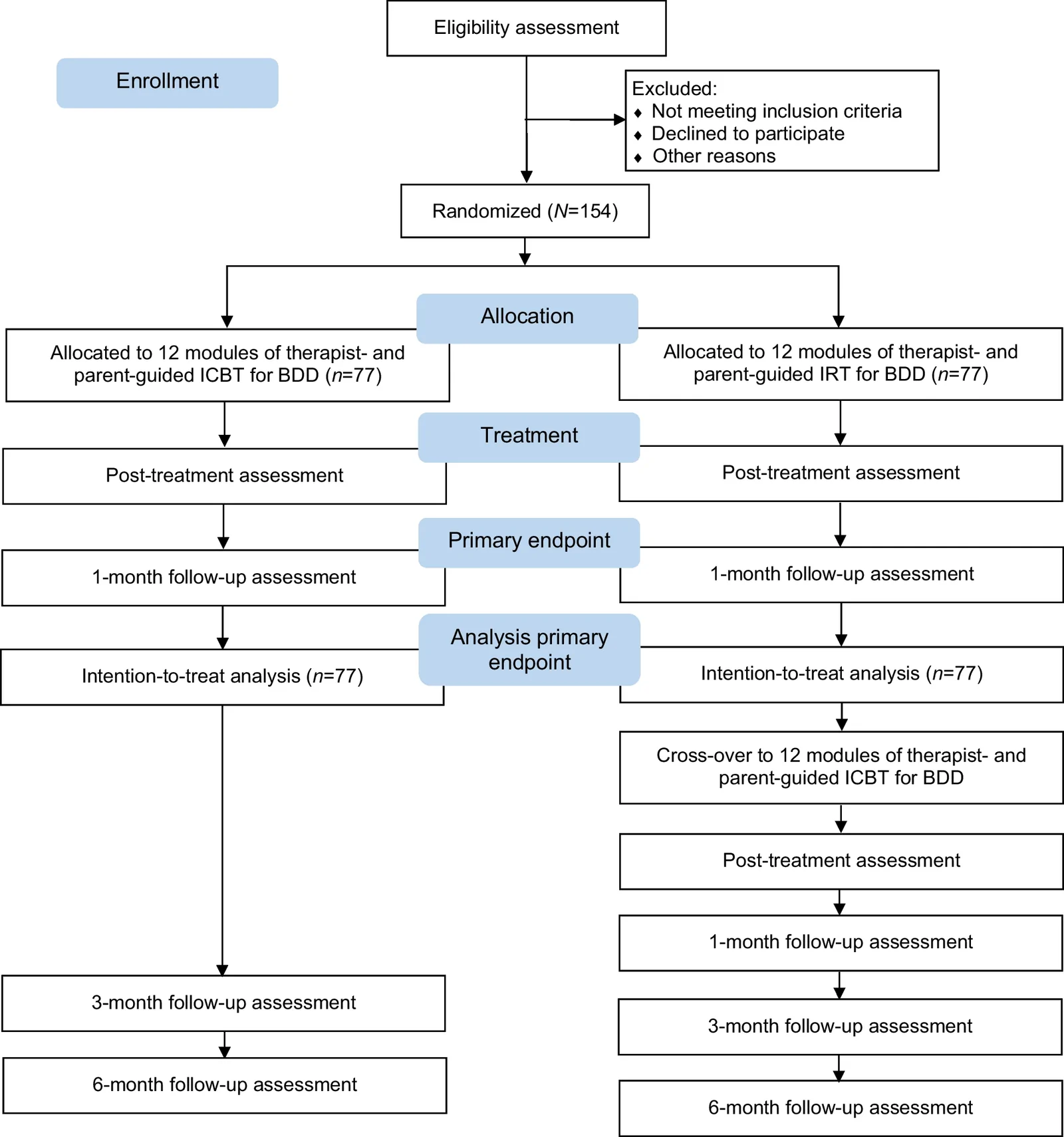
CONSORT 2025 flow of participants. Abbreviations: *BDD*, body dysmorphic disorder; *ICBT*, Internet-delivered cognitive-behavior therapy; *IRT*, Internet-delivered relaxation training

The study is a multisite, parallel-group, randomized controlled superiority trial for children and adolescents with BDD. Participants will be randomized to either 12 modules of therapist-guided ICBT or 12 modules of therapist-guided IRT for BDD. Participants will be assessed at baseline, mid-treatment, post-treatment, and 1 month, 3 months, and 6 months after the end of the treatment. The primary endpoint is the 1-month follow-up. At the primary endpoint, participants initially assigned to the IRT condition will be offered to cross-over to receive ICBT for BDD. Participants in either group who are still in need of further treatment after ICBT will be referred to their local services or other appropriate services. Participants in both arms will be followed up for 6 additional months after the end of the intervention (those initially randomized to IRT will be followed up regardless of whether they accept the cross-over or not). The 3- and 6-month follow-ups are naturalistic (i.e., uncontrolled). Figure 1 depicts the study design using the Consolidated Standards of Reporting Trials (CONSORT) 2025 [31] flow of participants.

The study sponsor is the Department of Clinical Neuroscience at Karolinska Institutet. Assessments and treatments will be conducted at three study sites: the Child and Adolescent Psychiatry Clinic for Obsessive-Compulsive Disorder (OCD) and Related Disorders (in Swedish, *BUP OCD och relaterade tillstånd*) in Region Stockholm, the Child and Adolescent Psychiatry Specialist Clinic (in Swedish, *BUP Specialmottagning*) in Västra Götalandsregionen, and the Child and Adolescent Psychiatry Clinic for Digital Treatment Skåne (in Swedish, *Barn- och ungdomspsykiatrimottagning digital behandling Skåne*) in Region Skåne. Each of the study sites will accept clinical referrals from their own regions but also from adjacent regions, according to the right to treatment principle. The trial will also accept self-referrals from anywhere in Sweden.

The trial is coordinated by the Stockholm study site, which is responsible for overall trial management, protocol implementation, investigator coordination, data collection procedures, and communication between participating sites. The local investigators at each site are responsible for participant recruitment, assessment, and delivery of study procedures at their respective sites.

### Participants

#### Eligibility criteria

The inclusion criteria for participation are (1) a primary diagnosis of BDD, based on the 5th edition of the Diagnostic and Statistical Manual of Mental Disorders (DSM-5) [32]; (2) a total BDD symptom severity score ≥24 on the Yale-Brown Obsessive-Compulsive Scale Modified for Body Dysmorphic Disorder, Adolescent version (BDD-YBOCS-A); (3) age between 12 and 17 years; (4) a minimum of one available parent/caregiver able to co-participate and support the adolescent throughout the treatment; and (5) regular access to a computer connected to the Internet and a mobile phone to receive text messages.

The exclusion criteria are (1) previous CBT for BDD for a minimum of five sessions with a qualified therapist within the 12 months prior to the inclusion assessment; (2) simultaneous psychological treatment for BDD or for any psychiatric comorbidity; (3) initiation, change of dosage, or cessation of any selective serotonin reuptake inhibitors (SSRIs) or antipsychotic drugs within the 6 weeks prior to the inclusion assessment; (4) a diagnosis of organic brain disorder, intellectual disability, psychosis, bipolar disorder, eating disorder, severe depression, or alcohol/substance dependence; (5) immediate risk to self or others requiring urgent medical attention or inpatient care, such as suicidality, or repeated self-injurious behaviors; (6) a documented suicide attempt in the last 12 months; (7) adolescent and parent/caregiver not able to read and communicate in Swedish; or (8) having a close relationship to an already included participant (e.g., sibling, cousin), to avoid being randomized into two different arms, with the risk of information “leaking” between the groups.

#### Recruitment

We will recruit through clinical referrals to the three participating clinics. All participants who are referred to either of the three clinics will, if eligible, be offered participation in the trial. We will also accept self-referrals from all over Sweden and will advertise the trial to clinics across the country, service user organizations, and the public via our website and social media.

All potential participating families will initially be contacted via telephone by a member of the research team, or in some cases approached face-to-face at the clinics for an initial screening. The main purpose of the preliminary screening is to briefly inform about the trial, conduct a preliminary screening of eligibility, and answer any questions that the families may have. If preliminarily eligible and interested, the potential participants will be booked for an inclusion assessment. Before the assessment, the potential participants will be provided with an age-appropriate information sheet, an informed consent form, and login information to fill in baseline questionnaires online. The potential participants and their parents/caregivers will sign the informed consent form prior to the inclusion assessment.

Inclusion assessments will be conducted face-to-face at the clinic by specialist clinicians working at one of the three recruitment sites. In some cases where in-person assessments are not feasible, the inclusion assessments may be conducted remotely via videoconferencing. For a diagnosis of BDD, it is necessary to rule out the presence of actual physical defects that are clearly noticeable (e.g., cleft palate, supernumerary fingers). If this is not possible or appropriate during the initial assessment (e.g., genitalia need to be explored), we will make a clinical referral to an appropriate service for an expert opinion.

Before inclusion, each assessor must discuss the potential participant with the corresponding local study coordinator or discuss it in the local weekly meetings held at the corresponding study site. If needed, the local study coordinator can also discuss the case further with the central study coordinator. If the assessor is uncertain about whether a potential participant is eligible, the principal investigator (Fernández de la Cruz) will be informed and have the final decision. If all eligibility criteria are met, the consent forms have been signed and reached the research team, and all baseline measures have been completed, the participant will be included in the trial. Then, participants will be randomized, be assigned a trial ID, and start treatment within 1 week from randomization.

### Randomization and allocation concealment

Participants will be randomized at a 1:1 ratio to one of the two interventions (ICBT or IRT) using randomly varying block sizes. Randomization will be conducted using an online randomization service (ALEA Clinical), set up and monitored by an independent clinical trials unit, the Karolinska Trial Alliance (KTA). The research team will not have access to the random allocation sequence. One or several researchers within the team (according to a task delegation list) will be responsible for the enrollment of participants, randomization, and assigning participants to treatments and therapists. Participants will be informed that they will be allocated to one of two psychological treatments for BDD, without providing specific details about the interventions or the study hypotheses.

### Interventions

#### Treatment format and therapist support

Both treatments are delivered over the Internet using BASS, a digital platform provided by the eHealth Core Facility at Karolinska Institutet that is also used as a data collection tool and database for storing the data. Both interventions are therapist-guided and consist of two separate sets of modules, one for the child/adolescent and one for the parent/caregiver, each with separate logins.

Table 1 contains an overview of the content of the interventions. Each intervention consists of 12 modules delivered over a maximum period of 14 weeks. During this time, participants (both the child/adolescent and the parent/caregiver) have regular contact with a therapist in the platform. The therapist will provide brief asynchronous support during the treatment, which includes feedback, answering questions, and sending reminders to complete the next module, if required. Communication during the treatment is possible via text messages in the online platform (resembling e-mail). The therapist usually logs in daily (on workdays, at least every 48 h). There are no specified limits to the therapist’s time. Telephone calls are possible if the participant or the therapist feel that they are needed. The participants are welcome to contact their therapist at any time, who will reply during office hours.

**Table 1:** Overview of the treatment modules for both child/adolescent and parents/caregivers in each condition

Module	Internet-delivered cognitive-behavior therapy arm	Internet-delivered relaxation training arm
1	Introduction and information about body dysmorphic disorder	Introduction and information about body dysmorphic disorder
2	Learning more about body dysmorphic disorder and set up treatment goals	Learning more about body dysmorphic disorder
3	What is exposure therapy?	What is anxiety?
4	Starting exposure practice	Starting relaxation practice: deep breathing
5	Focus on response prevention	Relaxation practice: arms, legs, and back
6	How body dysmorphic disorder affects family and school	Relaxation practice: head, jaw, neck, stomach, and chest
7	Body dysmorphic disorder thoughts and attention training	Relaxation practice: whole body
8	Full response prevention	Imagery (cognitive relaxation)
9	Exposure with response prevention practice	Relaxation with and without audio file in different positions
10	Exposure with response prevention practice	Repeating previously practiced relaxation exercises
11	Evaluating your goals and starting to look forward	Starting to look forward
12	Planning ahead	Planning ahead

In both interventions, the 12 modules are open at a pace of one module per week. After the post-treatment has finished, the child/adolescent and parent/caregiver can continue to access all treatment modules for the whole follow-up period (up to 6 months after treatment), but without therapist support.

The therapists delivering both the ICBT and IRT interventions are clinicians who will go through an extensive training including how to use the BASS platform, an interactive course on the assessment and treatment of pediatric BDD [33] (for the ICBT arm), and an online lecture on relaxation training and its application based on a structured manual [34] (for the IRT arm). Full details on the therapist training, supervision, and monitoring can be found in the full study protocol (Supplementary File 1).

The study interventions are not designed to be modified during the trial. Therefore, no criteria for intervention modification have been specified. Participants are free to discontinue the intervention at any time. Participants will not be withdrawn from the trial if they are inactive in treatment. If the child/adolescent states that they do not wish to continue their treatment, the parent/caregiver will still be encouraged to continue working with their separate modules. Participants and their parents/caregivers who do not complete the intervention will still be invited to the follow-up assessments (following intention-to-treat principles). Participants will be informed that it is helpful for the trial to collect outcome measures, even if they did not complete their treatment.

Concurrent psychological interventions for BDD or for any psychiatric comorbidity are not permitted at the time of inclusion. To ensure treatment stability, initiation, discontinuation, or dosage changes of SSRIs or antipsychotic medication within the 6 weeks preceding the inclusion assessment are not permitted. Participants may continue other types of psychotropic medications while participating in the trial. Information regarding any changes in psychological or pharmacological treatment during the trial will be recorded.

#### Internet-delivered cognitive-behavior therapy

The CBT treatment manual was initially developed in England for the first pediatric BDD RCT [5], further developed and tested at two specialist clinics [15], and subsequently digitalized to test its feasibility [29] in preparation for the current trial. The first part of the treatment includes psychoeducation about BDD, how avoidance and repetitive behaviors fuel and maintain appearance concerns, and strategies to resolve possible ambivalence toward psychological treatment, as many young people with BDD prefer cosmetic or surgical procedures, often due to the lack of insight into their mental health condition [1, 3]. The main goal of the treatment is to help the young person to stop avoiding anxiety-provoking situations (e.g., going to school or participating in other social situations) by undertaking exposure tasks and to stop doing unhelpful repetitive behaviors and rituals (e.g., excessive mirror checking, camouflaging, excessive use of makeup), known as response prevention (i.e., ERP). Every module also contains homework tasks to be completed between modules which mainly consist of ERP tasks based on the young person’s individual treatment goals.

Parents/caregivers are often involved in the patients’ rituals and avoidant behaviors (known as family accommodation), which may contribute to maintaining the BDD symptoms [35]. Incorporating parents/caregivers into the treatment has the potential to modify these unhelpful patterns. Parents/caregivers log in separately to receive psychoeducation, information on family accommodation and how to reduce it, and practical strategies to support their child/adolescent in the different ERP tasks.

#### Internet-delivered relaxation training

The active comparator is an adapted version of a relaxation training manual used in a trial for children with OCD [34]. The treatment is designed to match the above-described ICBT for BDD in all formal aspects (e.g., platform use, treatment length, therapist support, homework assignments) except for the module content, specifically ERP tasks.

The first part of the treatment includes psychoeducation about BDD, how anxiety is a major contributor to BDD symptoms, and presents the rationale that targeting and reducing anxiety will have a beneficial impact on BDD symptoms. The main goal of the treatment is to teach the young person to relax to cope with anxiety and appearance concerns. Further, because BDD symptoms are thought to be stress-sensitive (i.e., they may worsen in periods of anxiety or stress), participants are told that by learning to relax, they will experience fewer stress-related BDD symptoms through learning anxiety management strategies. The intervention mainly consists of different relaxation skills such as deep breathing, progressive muscle relaxation, and guided imagery relaxation. Every module also contains homework tasks that are meant to be completed between modules and mainly consist of practicing the relaxation learned in the online modules.

Parents/caregivers are also involved throughout the treatment and log in separately to receive psychoeducation about BDD, information on anxiety and how to reduce it, and strategies to assist their child/adolescent in the different relaxation tasks.

### Outcome measures

An overview of all outcome measures and assessment points is presented in Table 2. Additional details of the measures, including psychometric properties and time of application, are provided in the full study protocol available in Supplementary File 1. All clinician-rated measures will be collected face-to-face (or via videoconference or telephone, when face-to-face assessments are not feasible). All self- and parent-reported measures are administered via an online platform (BASS), which ensures automatic and complete entry of each measure into the trial database.

**Table 2:**
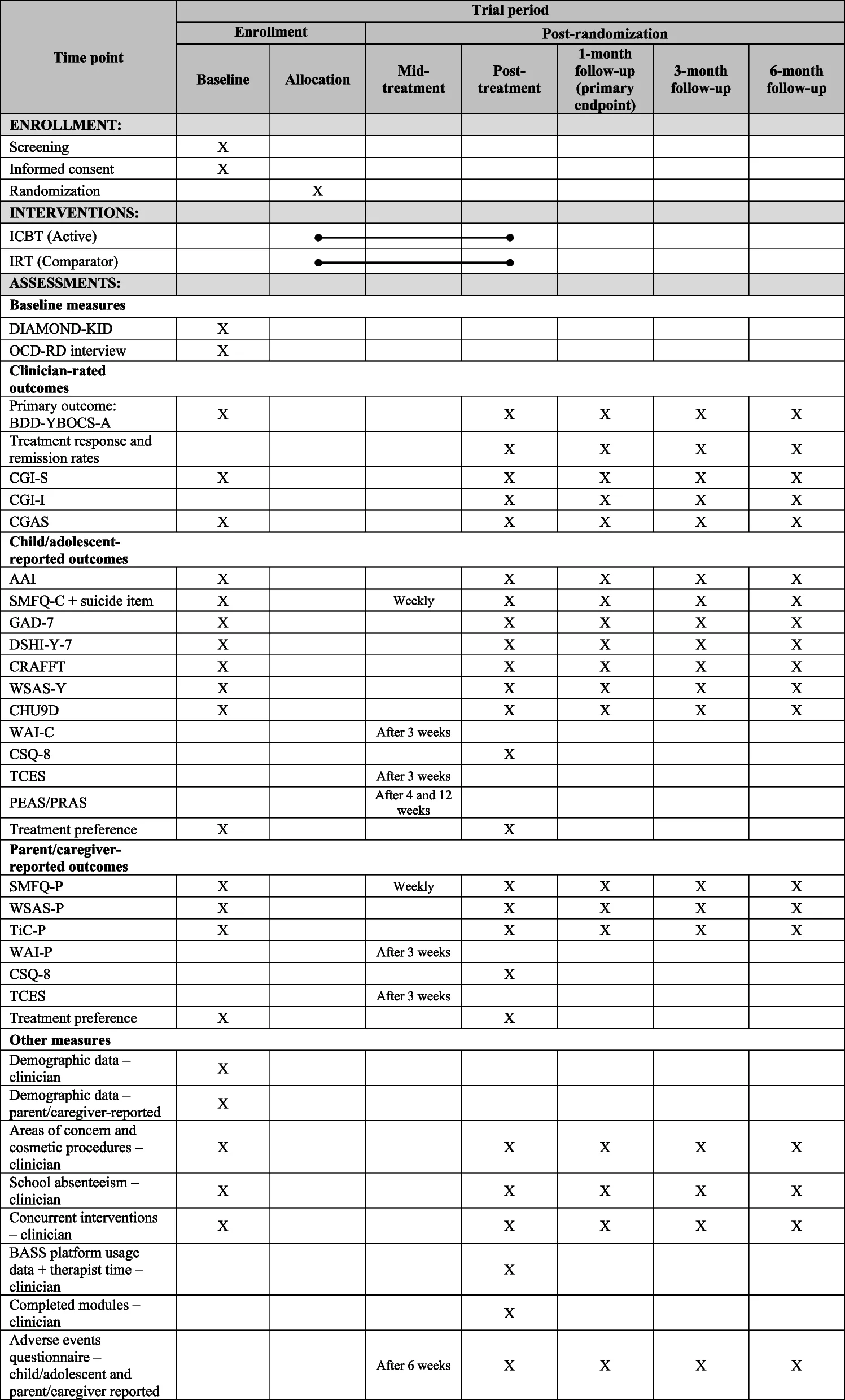
Overview of measures

Abbreviations: *AAI* Appearance Anxiety Index, *BDD-YBOCS-A* Yale-Brown Obsessive-Compulsive Scale Modified for Body Dysmorphic Disorder, Adolescent version, *CGAS *Children’s Global Assessment Scale, *CGI-I* Clinical Global Impressions Scale—Improvement, *CGI-S* Clinical Global Impressions Scale—Severity, *CHU9D* Child Health Utility 9D, *CRAFFT* acronym for the key words Car, Relax, Alone, Forget, Friends and Trouble, *CSQ-8* Client Satisfaction Questionnaire, *DIAMOND-KID* Diagnostic Interview for Anxiety, Mood, and OCD and Related Neuropsychiatric Disorders—Child and adolescent version, *DSHI* Deliberate Self-Harm Inventory Youth version, *GAD-7* Generalized Anxiety Disorder—7-item scale, *OCD-RD interview* obsessive-compulsive disorder and related disorders interview, *PEAS* Patient Exposure Adherence Scale, *PRAS* Patient Relaxation Adherence Scale, *SMFQ* Short Mood and Feeling Questionnaire, *TCES* Treatment Credibility and Expectancy Scale, *TiC-P* Treatment Inventory of Costs in Patients with Psychiatric Disorders, *WAI-C/P* Working Alliance Inventory—child and parent version, *WSAS-Y/P* Work and Social Adjustment Scale—youth and parent version

#### Baseline measures

Psychiatric diagnoses will be based on DSM-5 diagnostic criteria. A semi-structured interview based on DSM-5 criteria, the Diagnostic Interview for Anxiety, Mood, and OCD and Related Neuropsychiatric Disorders – Child and Adolescent Version (DIAMOND-KID) [36], will be used. Additional obsesive-compulsive related comorbidities not included in the DIAMOND-KID (e.g., olfactory reference syndrome) will be assessed using separate modeules from the Structured Clinical Interview for DSM-IV (SCID-I) [37].

#### Primary outcome measure

The primary outcome measure is the clinician-rated BDD-YBOCS-A [38, 39], measured at post-treatment and 1 (primary endpoint), 3, and 6 months after treatment completion. The BDD-YBOCS-A contains 12 items ranging from 0 to 4, including five questions on obsessions, five on compulsions, one about insight, and one to measure behavioral avoidance. The total BDD severity score ranges from 0 to 48, with higher scores denoting higher severity. The BDD-YBOCS-A has good psychometric properties [39] and is the gold standard in the BDD field to assess symptom severity. All assessors performing baseline evaluations and blinded assessors are extensively trained on the BDD-YBOCS-A by clinical experts at each site. Outcome assessors are trained to reliability (see the full study protocol in Supplementary File 1 for a complete description of the training procedures and establishing of the inter-rater reliability).

#### Secondary outcome measures

Treatment response and full or partial remission rates will be calculated at each follow-up point. Following current consensus in the field [40], treatment response will be defined as a reduction ≥30% on the BDD-YBOCS-A from baseline and full or partial remission will be defined as a total score ≤16 on the BDD-YBOCS-A.

Other secondary clinician-rated outcomes will include the Clinical Global Impressions Scale—Severity (CGI-S) and the Clinical Global Impressions Scale—Improvement (CGI-I), compared with baseline [41], as well as the Children’s Global Assessment Scale (CGAS) to assess global functioning of the young person [42]. All assessors will be trained in the use of the CGI-S, CGI-I, and CGAS and will rate them in addition to the BDD-YBOCS-A.

Secondary child/adolescent- and parent/caregiver-reported outcomes will include the self-reported Appearance Anxiety Inventory (AAI) to assess typical BDD cognitions and behaviors [43, 44]; the Short Mood and Feelings Questionnaire—child version (SMFQ-C) and parent version (SMFQ-P) to evaluate depressive symptoms [45], including an additional suicide item (only for the child/adolescent); the self-reported Generalized Anxiety Disorder—7-item scale (GAD-7) to assess anxiety [46]; the Deliberate Self-Harm Inventory—youth version (DSHI-Y-7) to assess the presence and frequency of non-suicidal self-injuries [47]; the self-reported CRAFFT (acronym for the key words Car, Relax, Alone, Forget, Friends, and Trouble) to identify substance use and substance use disorder [48, 49]; the Work and Social Adjustment Scale—youth version (WSAS-Y) and parent version (WSAS-P) to assess functional impairment [50, 51]; the Working Alliance Inventory—child version (WAI-C) and parent version (WAI-P) to measure perceived working alliance with the therapist [52]; the Client Satisfaction Questionnaire (CSQ-8) to assess treatment satisfaction in both the child/adolescent and their parents/caregivers [53]; the Treatment Credibility and Expectancy Scale (TCES) to measure treatment credibility or expectancy of treatment outcome [54]; the Patient Exposure Adherence Scale (PEAS) or Patient Relaxation Adherence Scale (PRAS)—according to group allocation—to measure patient adherence to either of the interventions [55]; and two items to assess treatment preference in both the child/adolescent and the parent/caregiver. Additionally, for the health economic evaluation, the child/adolescent will complete the self-reported Child Health Utility 9D (CHU9D), assessing quality of life [56], and the parent/caregiver will complete the Treatment Inventory of Costs in Patients with Psychiatric Disorders (TIC-P), assessing healthcare and societal resource use [57].

#### Additional measures

Additional measures will include demographic data, body areas of BDD-related concern, desire and/or undertaking of cosmetic procedures, school attendance, concurrent interventions, number of completed treatment modules, BASS platform usage data, and therapist time, including written messages in the platform and telephone calls.

### Safety procedures

A questionnaire will be administered to systematically assess adverse events throughout the trial. The questionnaire was developed by the research team and includes a checklist of expected adverse events based on previous trials as well as a free text box where other, not listed adverse events can be registered. Additionally, high scores on the SMFQ (depressive symptoms), with the additional suicide item, and the DSHI-Y-7 (self-harm) might also be indicators of adverse events. Scores ≥13 points on the SMFQ-C or SMFQ-P, or scores of 2 (“*I have had thoughts about death and of taking my life, but would never do it*”) or 3 (“*I have had thoughts about death and of taking my life*”) points on the suicide item on the SMFQ-C, will automatically raise a flag in the BASS system that will directly notify members of the research team to follow this up (via telephone) with the participant and their parents/caregivers. This will be recorded as an adverse event if the score is greater than their baseline score. The research team can also be notified about adverse events through direct (text or telephone) communication with the participant.

All adverse events will be documented at each of the sites by the corresponding local study coordinator in a specific log (including date, recorded clinical symptoms, and a brief description of the event), which in turn will be shared with the external monitor, the KTA. All adverse events will be summarized by number (frequencies and percentages), nature, severity, and whether they are expected or unexpected. The event will be classified as expected if it was listed as such in the study protocol (e.g., increased severity of BDD symptoms, lower mood, increased risk of self-harm, suicidal ideation, and/or suicide attempt; see Supplementary File 1 for the full list). If the event is not listed, then it will be classified as unexpected. If there are any serious adverse events (i.e., those resulting in death, life-threatening, requiring hospitalization, resulting in disability or incapacity, or considered medically significant by the principal investigator), the study coordinator and the principal investigator will judge whether these are related to the treatments provided. Events will be considered as potentially treatment-related up to the 6-month follow-up.

Appropriate action will be taken in the case of a serious adverse event, making sure the participant gets in contact with suitable health care services. If a serious adverse event occurs, the principal investigator will immediately notify the sponsor and the monitor; then they will work together to identify the extent of the serious adverse event and determine whether and what urgent safety measures are required.

### Blinding

All study personnel who can be blinded to study aims/hypotheses and group allocation will be blinded. Assessors conducting post-treatment and follow-up assessments will be external to the research team and blinded to study aims/hypotheses and treatment allocation for the full duration of the trial. The assessors will not receive any information about the study design, objectives, or treatment arms to maximize blinding integrity. At each follow-up assessment, participants will be reminded by their assessor not to reveal details about their treatment. Table 3 shows which individuals are blinded to the study aims/hypotheses and/or group allocation.

**Table 3:** Individuals involved in the trial who are blinded to study aims/hypothesis and/or group allocation

Individuals involved in the trial	Blinded to study aims/hypotheses	Blinded to group allocation
Principal investigator	No	Yes
Co-investigators	No	Yes
Study coordinator	No	No
Trial therapists	No	No
Study participants	Yes	No
Outcome assessors	Yes	Yes
Data managers	No	Yes
External monitors (Karolinska Trial Alliance)	No	No
Statistician	Yes	Yes
Health economist	No	Yes

The primary analysis will be performed after the trial’s final participant has finished their 1-month follow-up assessment (primary endpoint). When the statistical analyses are completed, and before unblinding, different versions of the abstract will be written, each reaching different conclusions (e.g., intervention A is superior to intervention B, or vice versa). After unblinding, the correct version of the abstract will be used in the final report.

In case of a medical emergency, participants will be encouraged to immediately seek appropriate healthcare and to inform their therapist, who in turn will notify the study coordinator. The local study coordinator will oversee following up the incident. This procedure can be done without unblinding the outcome assessors and, therefore, an emergency unblinding system is not required.

### Patient and public involvement

The feasibility trial preceding the current RCT [29] collected quantitative data on feasibility and preliminary efficacy of the active intervention, as well as detailed qualitative feedback from participating adolescents, their parents, and treating clinicians. Based on this feedback, adjustments to optimize the intervention (e.g., changes to increase participant motivation and improve adherence to the treatment modules) were implemented.

Additionally, the RCT includes a Research Steering Group of young people with BDD and their families. The Research Steering Group provided advice regarding study design, trial conduct, participant engagement, and dissemination activities. This group was recruited from the pool of participants in the feasibility trial and from our specialist clinics. The Research Steering Group also includes representatives from the Swedish service user organizations *Riksföreningen Dysmorfofobi*—*BDD* (the Swedish Body Dysmorphic Disorder Association) and *OCD-förbundet* (the Swedish OCD Association). They have contributed to the optimization of the study design and the optimization of the treatments used in both arms of the RCT. Regular meetings are scheduled to offer updates on the running of the trial and to discuss specific issues related to study implementation and recruitment (e.g., how to increase outreach and detection of BDD cases in youth across the country in order to speed up the recruitment pace). The Research Steering Group members receive compensation for their participation.

### Power analysis

Between-group effect sizes in an RCT comparing face-to-face CBT with an active control in youth with BDD [5] and an RCT comparing ICBT with an active control in adults with BDD [26] were *d* = 1.13 and *d* = 0.94, respectively. Other pediatric ICBT trials similar to ours have reported smaller effect sizes. For example, an ICBT study of adolescent OCD [58] and an ICBT study of adolescent social anxiety disorder [59] reported between-group effect sizes of *d* = 0.69 and *d* = 0.64, respectively. Similarly, an adult BDD trial with an active control condition reported an effect size of *d* = 0.76 [60]. Thus, we have powered our study to be able to detect at least a moderate effect size of *d* = 0.50. A sample size of 128 participants was calculated to be sufficient with a power of 80%, with a 2-tailed *α* = 0.05. An additional 20% of participants will be added to account for potential dropouts. Hence, we aim to recruit a total of 154 participants (77 per group).

### Statistical analysis

Statistical analyses relating to the clinical efficacy and 6-month durability aims will be conducted in-house by the study coordinator at the Stockholm site (author Birovecz) and then independently replicated by personnel at the Biostatistics Core Facility at Karolinska Institutet (under the direction of author Bottai). The economic evaluation will be conducted under the supervision of author Andersson at Karolinska Institutet, who will also be blinded to group allocation. A full statistical and health economic analysis plan (see Supplementary File 2) will guide the statistical and health economic analyses. This plan will be finalized before the last participant has reached the primary endpoint. In the possible case that any information regarding statistical analyses would differ from this protocol, the latest version of the statistical and health economic analysis plan will be considered the original source to guide analytical decisions.

The demographic information collected at baseline will be summarized and presented in a table, by randomization arm. Categorical variables will be reported as counts and percentages. Continuous variables will be summarized as means, medians, and interquartile ranges, as appropriate. According to CONSORT recommendations, no statistical tests will be performed to assess baseline differences between study arms [31]. No interim analysis is planned.

#### Primary outcome analysis

The main analysis will follow a prespecified intention-to-treat statistical analysis plan, which will be finalized and preregistered prior to unblinding. The primary outcome variable is the BDD-YBOCS-A total severity score at the primary endpoint (1-month follow-up). All the randomized participants will be included in a linear mixed-effect model to estimate the difference between group means over time. Linear mixed models use all available data, can properly account for correlation between repeated measurements on the same subject, have greater flexibility to model time effects, and can handle missing data. The model will include fixed effects of time (baseline, post-treatment, and 1-month follow-up), treatment group (ICBT or IRT), site (Region Stockholm, Västra Götalandsregionen, or Region Skåne), and interaction effects of group by time, group by site, site by time, and group by site by time, as well as random intercept to account for individual differences. If the effects of site are not statistically significant, this variable will be dropped from the model to obtain a more parsimonious model. The primary source of inference will be the between-group difference at the primary endpoint (1-month follow-up), estimated from the group by time interaction. Additionally, between-group effect sizes will be estimated using Cohen’s *d*.

#### Secondary outcomes analysis

Secondary outcome variables will be analyzed using the same modeling framework used for the primary outcome variable, although the study is not powered to analyze these outcomes or moderation effects on the primary outcome. Dichotomous outcomes will be analyzed using logistic regression. The results will be presented as estimates or odds ratios (with respective 95% confidence intervals [CIs] and *p *values), as appropriate. We will also compare the proportions of treatment responders and full or partial remitters in each group with logistic regression and odds ratio (95% CI). Between-group effect sizes will be estimated using Cohen’s *d*.

#### Long-term follow-up analysis

All trial participants will be naturalistically followed up and assessed up to 6 months after the end of the treatment. Linear mixed-effects models will be implemented for the primary and secondary outcome measures. As for the primary outcome, for each secondary outcome measure, the model will include fixed effects of time (three time points: 1-month follow-up, 3-month follow-up, 6-month follow-up) and site, as well as a time by site interaction, and subject effects as a random intercept factor to account for the variances between and within participants. As above, site will be dropped from the model if its effects are not significant.

#### Health economic analysis

For the reporting of the health economic evaluation, we will follow the Consolidated Health Economic Evaluation Reporting Standards (CHEERS) 2022 guidelines [61]. The health economic evaluation will compare ICBT and IRT at the primary endpoint (1-month follow-up) from three different perspectives, with gradual broadening of the cost calculations for each perspective:

1. The first perspective is the health organization payer perspective, which includes the direct costs of running the interventions for the clinic. This comprises personnel costs such as therapist time, administration time, IT platform maintenance, and other overhead costs.
2. The second perspective will additionally include other health care resource use outside the clinic for the participating children/adolescents, such as costs for medical appointments (outside the trial) and medications.
3. The third perspective will further comprise all societal costs, including productivity losses for the children/adolescents related to absenteeism and presenteeism from school and leisure activities, and productivity losses for the parents due to absenteeism from work.

The TIC-P [57] used in the current trial has been adapted for young people and parents/caregivers. It will be administered at each assessment point (except during the treatment) to collect information on frequencies of resource use and productivity losses. Resource use costs will be estimated by multiplying frequencies by national Swedish tariffs and market prices. Total costs for each group will be aggregated over the trial period.

For each of the three perspectives above, we will conduct two types of analyses. First, a cost-utility analysis using quality-adjusted life years (QALYs) as the outcome. Second, a cost-effectiveness analysis using responder rate as the outcome.

Health gains in terms of QALYs will be estimated using CHU9D scores. CHU9D scores will be transformed into individual health-related quality of life scores, which will be used to estimate QALYs over the trial period, using the area under the curve method. We will compare costs and health outcomes between the two intervention arms over time using generalized linear models.

To ascertain whether the intervention is cost-effective, we will calculate an incremental cost-effectiveness ratio, as the ratio between the difference in costs and the difference in QALYs or responders between interventions, for each costing perspective and express them as cost per additional QALY and cost per responder. We will explore uncertainty around the cost and outcome estimates and perform relevant sensitivity analyses.

### Quality control

The trial will be conducted according to Good Clinical Practice (GCP) principles. All trial staff will attend GCP training. Quality procedures, including documentation and randomization, will be set up with the help of the KTA. GCP documents such as source data and task delegation lists will be established. The KTA will perform case-by-case monitoring of informed consent, eligibility criteria, data entry (i.e., 100% source data verification of the primary outcome measure, the CGI-S, CGI-I, and CGAS at baseline and the primary endpoint, and 10% spot check source data verification of these measures at all other time points), and adverse events. Any discrepancies identified during monitoring will be documented and resolved by the study team.

Study data are collected both manually (paper case report forms; CRFs) and digitally (child/adolescent- and parent/caregiver-reported questionnaires completed via the Internet directly into the trial database in BASS). The CRFs will not bear the participant’s name. Instead, the trial identification number will be used for identification. CRFs will be stored securely (separate from the code key) in a locked file cabinet at each of the three clinics. Data from the CRFs will consecutively be entered manually into BASS by a member of the research team at each of the sites. Clinical data will be quality-checked at the time of collection. Data entered into the BASS database are validated to predefined value ranges and consistency checks, and the primary outcome data are monitored by the KTA.

Weekly meetings at each site with the local study team, as well as weekly meetings of the study coordinators at each of the sites, are conducted to ensure that trial procedures are being followed, including treatment fidelity and adherence to the protocol by the therapists guiding the participants’ treatments.

No formal Data Monitoring Committee has been established for this trial. Given the low-risk nature of the intervention, independent oversight is instead provided through external monitoring by the KTA.

### Ethics

The Swedish Ethical Review Authority approved the study (original application reference number 2023–02193-01 and amendment reference number 2024–02036-02). The original ethical approval document and an automatically generated English translation of this document are available in Supplementary Files 3 and 4. Verbal information about the study and written informed consent forms are provided to the child/adolescent and all legal guardians prior to baseline assessment. All participant families are volunteers competent to provide informed consent. Participants may withdraw from the trial at any time. The informed consent form includes a provision informing participants that de-identified data collected during the trial may be used in future research projects and may be shared with other researchers in accordance with applicable ethical approvals, legal requirements, and data protection regulations. No biological specimens will be collected.

The interventions pose a small risk to participants. Both interventions (ICBT and IRT) differ from their respective face-to-face versions only in the format and mode of delivery but still contain the same evidence-based components. While there is some preliminary evidence to suggest that ICBT may be efficacious for individuals with BDD [16, 26, 28, 29], at present, there is no evidence that it will be superior to IRT in reducing BDD symptom severity. Thus, the trial fulfills the principle of equipoise. Nonetheless, our hypothesis is that ICBT may indeed be superior to IRT. For that reason, all individuals randomized to the active comparator (IRT) will be offered crossover after the controlled period ends at the 1-month follow-up.

Patient safety is of high priority in this study. Participants complete measures of depression and suicidality weekly, allowing for these symptoms to be monitored throughout the intervention. A thorough diagnostic and mental health status examination before enrollment of participants will prevent patients with more immediate needs or risks from being included in the study. Children and adolescents who have recently (during the previous year) attempted suicide will be excluded to reduce the risk of suicide attempts and increase patient safety during the trial. These patients will be referred to other services or offered alternative treatment at the clinic, as appropriate.

This study will be conducted within the Swedish regular health care system, and all participants will be enrolled as patients at one of the three clinics. As patients, they will be followed according to the ordinary clinical routines and will continue to have access to standard clinical care during and after trial participation. We will also have clinical weekly meetings to discuss participants. Specific procedures will be in place to handle potential acute situations (e.g., self-harm, suicidality) and clinical personnel will be able to quickly refer participants in need of more help to their local mental health care services or emergency services, if needed. No additional ancillary or post-treatment care arrangements are planned. Participants are covered by the standard patient insurance and compensation systems that apply within the Swedish health care system.

Participants may worry about computer safety and confidentiality. To prevent this, families will receive information about the risks and precautions that are being taken when using communication technology (e.g., encrypted server technology and double authentication with password and text message code). Swedish and international rules and regulations will be followed. Our research team has extensive experience and knowledge of the approval processes, systems, and good practice guidelines for clinical research in Sweden and internationally.

Each participant will be assigned a unique trial identification number at the point of inclusion. This number will be recorded on all paper datasheets and in the electronic trial database. All data will be kept secure and maintained in accordance with the requirements of GCP regulations. The Karolinska Institutet Electronic Laboratory Notebook (ELN) will be used to log all significant decisions during the trial.

Overall, the risks for the participants are assessed as limited and temporary. The potential benefits for the participants and the scientific value of the study far outweigh the potential risks of participation.

### Dissemination

The trial will be reported according to the CONSORT [31] and the CHEERS [61] statements. We plan to publish one or two primary papers. The main paper will report on the efficacy of the interventions (up to the 1-month follow-up [primary endpoint]) and follow-up data (from the 1-month follow-up to the 6-month follow-up). The cost-effectiveness results may be included in the main report or published separately. Additional papers may include analysis of predictors and moderators of treatment outcome. All manuscripts will be submitted to general or specialist psychological or medical science journals. Results will also be presented at scientific conferences, communicated to the trial’s Research Steering Group, and disseminated among service user organizations and the general public.

## Discussion

To our knowledge, this will be the largest RCT conducted in the field of BDD to date and only the second focusing on adolescents. For the first time, we will also conduct a health economic evaluation of the active intervention in individuals with BDD in this age group, which will allow us to inform policymakers. Furthermore, we have recruited a group of service users and their parents, as well as representatives from service user organizations to our Research Steering Group to participate in the research process, which is crucial to ensure that the digital interventions are acceptable and ecologically valid, fit with needs and lifestyles, and to tackle issues such as nonadherence. We are also contributing to increasing awareness of BDD in clinical settings and in the general population (by partnering with service user organizations that have a broad outreach), which we hope will contribute to earlier detection of the disorder. If ICBT for adolescent BDD is proven efficacious, safe, durable, and cost-effective, the goal is to implement it in Swedish regular healthcare. The hope is to increase access to evidence-based treatment for children and adolescents with BDD, hence reducing the inequalities associated with limited access to specialist care.

The results will be interpreted considering the potential limitations of the study design. First, our study uses an active comparator (i.e., IRT) that may also be somewhat effective in the reduction of BDD symptoms. Hence, in a scenario where our superiority hypothesis is rejected, it will be difficult to attribute this to a lack of efficacy of the active intervention (i.e., ICBT). However, we decided against including a pure waitlist control group on ethical and scientific grounds. Second, our eligibility criteria exclude participants with a number of acute or severe comorbidities, including conditions that complicate differential diagnosis with BDD (e.g., eating disorders), as well as individuals with recent suicide attempts, which may limit the generalizability of the results. However, the study is designed to ensure patient safety during the trial, and we are not excluding other comorbidities (e.g., autism spectrum disorders) that are generally excluded in many clinical trials.

## Trial status

The trial was prospectively registered on ClinicalTrials.gov on 16 February 2024 (https://clinicaltrials.gov/study/NCT06262412; title: Internet-delivered cognitive-behavior therapy for child and adolescent body dysmorphic disorder). The first trial participant was randomized on 22 February 2024, when protocol version 1.1 was in use. The current full study protocol in use is version 1.5, dating 25 May 2026. See Supplementary File 1 for the current version of the protocol and a full description of all protocol changes. The protocol will be further amended as needed, and reasons for the changes will be detailed, in consecutively numbered protocol versions. Any important protocol modifications (e.g., changes to study objectives, eligibility criteria, interventions, outcomes, sample size, or analyses) will be communicated promptly to all relevant parties. This includes the sponsor, the external monitor, the Swedish Ethical Review Authority, ClinicalTrials.gov, and investigators. Updates will also be reflected in the trial registration and reported in subsequent trial publications as appropriate. As of 21 August 2026, 100 out of 154 participants have been recruited. According to the current time plan, recruitment of the last participant will be completed in November 2027. The final participant is expected to complete treatment in February 2028, reach the primary endpoint in March 2028, and the 6-month follow-up assessment in August 2028.

## Supplementary Information


Additional file 1: Supplementary Table 1. SPIRIT 2025 checklist.Additional file 2: Supplementary File 1. Full study protocol, version 1.5.Additional file 3: Supplementary File 2. Statistical and health economic analysis plan, version 1.0.Additional file 4: Supplementary File 3. Ethical approval documents in Swedish (original).Additional file 5: Supplementary File 4. Ethical approval documents in English (automatically generated translation).

## Data Availability

No datasets were generated or analysed during the current study.

## Abbreviations

AAI: Appearance Anxiety Inventory
BDD: Body dysmorphic disorder
BDD-YBOCS-A: Yale-Brown Obsessive-Compulsive Scale Modified for Body Dysmorphic Disorder, Adolescent version
CBT: Cognitive-behavior therapy
CGAS: Children’s Global Assessment Scale
CGI-I: Clinical Global Impressions–Improvement scale
CGI-S: Clinical Global Impressions–Severity scale
CHEERS: Consolidated Health Economic Evaluation Reporting Standards
CHU9D: Child Health Utility 9D
CONSORT: Consolidated Standards of Reporting Trials
CRAFFT: Car, Relax, Alone, Forget, Friends, and Trouble
CSQ-8: Client Satisfaction Questionnaire
DIAMOND-KID: Diagnostic Interview for Anxiety, Mood, and OCD and Related Neuropsychiatric Disorders - Child and adolescent version
DSHI-Y-7: Deliberate Self-Harm Inventory–Youth version
DSM-5: Diagnostic and Statistical Manual of Mental Disorders, 5th edition
ELN: Electronic Laboratory Notebook
ERP: Exposure with response prevention
GAD-7: Generalized Anxiety Disorder–7-item scale
GCP: Good clinical practice
ICBT: Internet-delivered cognitive-behavior therapy
IRT: Internet-delivered relaxation training
KTA: Karolinska Trial Alliance
OCD: Obsessive-compulsive disorder
PEAS: Patient Exposure Adherence Scale
PRAS: Patient Relaxation Adherence Scale
QALY: Quality-adjusted life year
RCT: Randomized controlled trial
SCID-I: Structured Clinical Interview for DSM-IV
SMFQ-C: Short Mood and Feelings Questionnaire–Child version
SMFQ-P: Short Mood and Feelings Questionnaire–Parent version
SPIRIT: Standard Protocol Items: Recommendations for Interventional Trials
SSRI: Selective serotonin reuptake inhibitor
TCES: Treatment Credibility and Expectancy Scale
TIC-P: Treatment Inventory of Costs in Patients with Psychiatric Disorders
WAI-C: Working Alliance Inventory–Child version
WAI-P: Working Alliance Inventory–Parent version
WSAS-P: Work and Social Adjustment Scale–Parent version
WSAS-Y: Work and Social Adjustment Scale–Youth version
