# Supplementary material for: Efficacy and cost-effectiveness of Internet-delivered cognitive-behavior therapy for children and adolescents with body dysmorphic disorder: study protocol for a randomized controlled trial
Source: Trials. 2026 Aug 31;27:557. doi: 10.1186/s13063-026-09974-z (PMC13527960; doi:10.1186/s13063-026-09974-z)
Supplement: Supplementary file 1 [file 13063_2026_9974_MOESM2_ESM.pdf]

Karolinska Institutet

STUDY PROTOCOL:

Internet-delivered cognitive-behaviour therapy for child and adolescent body dysmorphic disorder

Long Title of the Trial	Clinical efficacy and cost-effectiveness of Internet-delivered cognitive-behaviour therapy for children and adolescents with body dysmorphic disorder: a randomised controlled trial
Short Title of the Trial	Internet-delivered cognitive-behaviour therapy for child and adolescent body dysmorphic disorder
Version and Date of Protocol	Version 1.5, 2026-05-25
Sponsor	Karolinska Institutet, Department of Clinical Neuroscience (CNS), Stockholm, Sweden
Funder(s)	Region Stockholm (ALF Medicin), Region Skåne (ALF Projekt), Folksams forskningsstiftelse, the International OCD Foundation, the Jane och Dan Olssons Stiftelse för Vetenskapliga ändamål, Centre for Psychiatry Research at Region Stockholm/Karolinska Institutet, Fredrik och Ingrid Thurings stiftelse, Crafoordska stiftelsen, Stiftelsen Söderström - Königska sjukhemmet, Stiftelsen Lindhaga, and Drottning Silvias Jubileumsfond
Trial Registration Number	ClinicalTrials.gov: NCT06262412
Ethical Review	Swedish Ethical Review Authority approval number: 2023-02193-01 and amendment approval number: 2024-02036-02
Sites	BUP OCD och relaterade tillstånd<br>Gävlegatan 22, Plan 8<br>113 30 Stockholm<br><br>BUP Specialmottagning<br>Sahlgrenska Universitetssjukhuset<br>416 85 Göteborg<br><br>Barn- och ungdomspsykiatrimottagning digital behandling Skåne<br>Per Albin Hanssons väg 41<br>205 02 Malmö
Principal Investigator	Dr Lorena Fernández de la Cruz<br>Department of Clinical Neuroscience<br>Gävlegatan 22, Plan 8, 113 30 Stockholm, Sweden<br>lorena.fernandez.de.la.cruz@ki.se

	+46 76-847 79 99
Sponsor Representative	Mats Olsson<br>Department of Clinical Neuroscience<br>Tomtebodavägen 18A floor 5, 171 77 Stockholm<br>mats.j.olsson@ki.se<br>+46 8-524 824 64

VERSION HISTORY

Version number	Version date	Reason for Change
1.0	2023-03-31	First version of the study protocol. Submitted to the Swedish Ethical Review Authority.
1.1	2024-01-11	A number of minor changes have been made to the protocol. These include:<br>• We slightly modified the criteria for training of therapists. Experienced therapists who are already working at an OCD specialist clinic and have worked with BDD patients for at least a year or have treated at least 3 patients with BDD will not be required to complete the Internet-based training course.<br>• We elaborated on the clinical study routines about meetings to discuss participants. During the duration of the trial, weekly meetings will be held together with the local study coordinators at each site and the central study coordinator to discuss cases.<br>• We elaborated on the clinical routines prior to inclusion. Before inclusion, each assessor must discuss the case with the local study coordinator or at the weekly meetings at the corresponding study site. If needed, the study coordinator of a site can discuss the case further with the central study coordinator.<br>• We amended an error in the Measures section of the protocol: the WSAS-P will not be administered after 6 weeks.<br>• At post-treatment and all follow-up assessments, we will drop the questions in the DSHI-Y-7 asking about the last 12 months, only the questions about self-harm during the last month will be asked.<br>• Spelling errors were corrected and some sentences were slightly reworded to improve clarity. This did not affect the content of the protocol.
1.2	2024-03-19	ClinicalTrials.gov registration number added to the cover page.<br>Clarification of exclusion criterion nr 6 regarding previous suicide attempts.
1.3	2024-12-10	Minor updates to the protocol:<br>• The ethical approval number and an amendment approval number have been added to the cover page.<br>• New funders have been added to the cover page.

		• The participant flowchart has been updated.<br>• Baseline assessor and blinded rater training requirements have been clarified.<br>• The monitoring of the communication between participants and therapists during the trial period has been further specified.<br>• A small error has been corrected about the scoring of the SMFQ.<br>• Spelling errors were corrected and minor edits were done to improve clarity.
1.4	2026-01-13	Minor changes have been made to the protocol, including:<br>• "Control intervention/condition" has been reworded to "active comparator" to highlight the equipoise between the interventions.<br>• An error has been corrected in Table 1. The OCD-RD interview is only applied at baseline.<br>• In Table 2, we have corrected that data managers are blinded to study aims/hypotheses and have removed the conclusion maker row.<br>• In section 6.3 it has been corrected the way that intervention modules are presented to the participants. One new module is opened each week.<br>• Minor wording changes have been made to the description of the IRT intervention in section 6.3.2.<br>• Additional funders have been added to page 1 and section 13.<br>• Minor edits were made to improve clarity.
1.5	2026-05-25	The semi-structured interview to evaluate psychiatric disorders at the inclusion assessment has been changed from the MINI-KID (Mini International Neuropsychiatric Interview for Children and Adolescents) to the DIAMOND-KID (Diagnostic Interview for Anxiety, Mood, and OCD and Related Neuropsychiatric Disorders, child and adolescent version).

SIGNATURE

Protocol version number: 1.5

Lorena Fernández de la Cruz, Principal Investigator

Date: __May 25th 2026_______________

Signature: 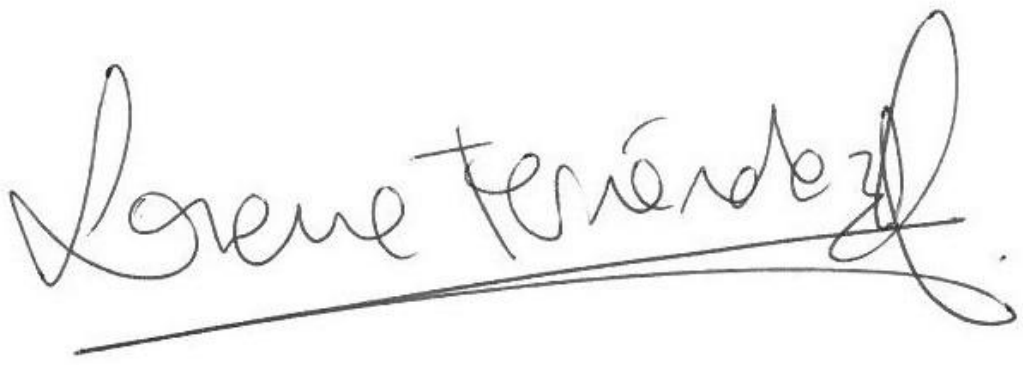

TABLE OF CONTENTS

1 TRIAL PERSONNEL 8
2 SUMMARY 10
3 INTRODUCTION 13
4 OBJECTIVES 14
5 PROJECT DESCRIPTION 15
5.1 DESIGN 15
5.2 STUDY SITES 17
5.3 POWER ANALYSIS 17
5.4 INCLUSION AND EXCLUSION CRITERIA 17
5.4.1 INCLUSION CRITERIA 17
5.4.2 EXCLUSION CRITERIA 18
5.5 MEASURES 19
5.5.1 BASELINE MEASURES 21
5.5.2 PRIMARY OUTCOME 21
5.5.3 SECONDARY CLINICIAN-RATED OUTCOMES 22
5.5.4 SECONDARY CHILD/ADOLESCENT AND CAREGIVER/PARENT-REPORTED OUTCOMES 23
5.5.5 OTHER MEASURES 26
6 PROCEDURE 28
6.1 RECRUITMENT 28
6.1.1 RECRUITMENT RATE 28
6.1.2 SCREENING AND RECRUITMENT PROCEDURES 28
6.2 RANDOMISATION, ENROLMENT, AND MASKING 30
6.3 TREATMENT 31
6.3.1 ICBT FOR CHILD AND ADOLESCENT BDD 32
6.3.2 INTERNET-DELIVERED RELAXATION TREATMENT 32
6.3.3 THERAPIST TRAINING, SUPERVISION, AND MONITORING 33
6.4 END OF TRIAL 34
6.5 PARTICIPANT WITHDRAWAL FROM TRIAL 34
6.6 DISCONTINUATION OF TRIAL 35
6.7 QUALITY CONTROL 35
7 DATA MANAGEMENT 35
7.1 DATA COLLECTION AND HANDLING 36
7.1.1 BASS 36

7.1.2 VIDEOCONFERENCE SOFTWARE 37
8 STATISTICAL ANALYSIS 37
8.1 PRIMARY OBJECTIVE: CLINICAL EFFICACY OF ICBT FOR BDD 37
8.2 SECONDARY OBJECTIVE 1: 6-MONTH DURABILITY OF THE TREATMENT EFFECTS 38
8.3 SECONDARY OBJECTIVE 2: HEALTH ECONOMIC EVALUATION 38
8.4 INTERIM ANALYSIS 39
9 ADVERSE EVENTS 39
9.1 ADVERSE EVENTS AND SERIOUS ADVERSE EVENTS 39
9.2 EXPECTED ADVERSE EVENTS 40
9.3 RELATED ADVERSE EVENTS 40
9.4 HANDLING OF ADVERSE EVENTS 41
10 DATA SHARING 41
11 ETHICAL CONSIDERATIONS 42
12 IMPLICATIONS 43
13 FUNDING 43
14 PUBLICATION PLANS 43
REFERENCES 44

1 TRIAL PERSONNEL

Principal Investigator:	Dr Lorena Fernández de la Cruz<br>Karolinska Institutet<br>Department of Clinical Neuroscience<br>Gävlegatan 22, Plan 8 113 30 Stockholm<br>lorena.fernandez.de.la.cruz@ki.se<br>+46 76 847 79 99
Sponsor representative:	Professor Mats Olsson, PhD<br>Karolinska Institutet<br>Department of Clinical Neuroscience<br>Tomtebodavägen 18A floor 5, 171 77 Stockholm<br>mats.j.olsson@ki.se<br>+46 8-524 824 64
Statistician:	KI Biostatistics Core Facility<br>Karolinska Institutet<br>Institute of Environmental Medicine<br>Box 210, 171 77 Stockholm
Co-investigators	Professor David Mataix-Cols<br>Karolinska Institutet<br>Department of Clinical Neuroscience<br>Gävlegatan 22, Plan 8 113 30 Stockholm<br>david.mataix.cols@ki.se<br>+46 72-398 68 89
	Dr Karin Melin<br>Gothenburg University<br>Institute of Health and Care Sciences<br>Sahlgrenska Universitetssjukhuset, BUP Specialmottagning<br>416 85 Götenborg<br>karin.a.melin@vgregion.se<br>+45 73-049 43 48
	Professor Eva Serlachius<br>Lund University<br>Department of Clinical Sciences<br>Barn- och ungdomspsykiatri (BUP)<br>Baravägen 1, Forskningsenheten 221 85 Lund<br>eva.serlachius@med.lu.se<br>+46 70-715 52 32
	Dr Kristina Aspvall<br>Karolinska Institutet<br>Department of Clinical Neuroscience<br>Gävlegatan 22 floor 8, 113 30 Stockholm<br>kristina.aspvall@ki.se

+46 73-909 41 33
Dr Erik Andersson
Karolinska Institutet
Department of Clinical Neuroscience
Nobels Väg 9, 171 77 Stockholm
erik.m.andersson@ki.se
+46 73-671 63 35

Maria Silverberg-Mörse
BUP Forsknings- och utvecklingscentrum
Region Stockholm
Gävlegatan 22 floor 8, 113 30 Stockholm
maria.silverberg-morse@regionstockholm.se
+46 81-235 22 22

Frida Wickberg
BUP Forsknings- och utvecklingscentrum
Region Stockholm
Gävlegatan 22 floor 8, 113 30 Stockholm
frida.wickberg@regionstockholm.se
+46 81-235 22 11

Daniel Rautio
Karolinska Institutet
Department of Clinical Neuroscience
Gävlegatan 22, Plan 8 113 30 Stockholm
daniel.rautio@ki.se
+46 73-057 78 01

Anita Birovecz
Karolinska Institutet
Department of Clinical Neuroscience
Gävlegatan 22, Plan 8 113 30 Stockholm
anita.birovecz@ki.se
+46 72-166 95 44

Dr Per Andrén
Lund University
Department of Clinical Sciences
Barn- och ungdomspsykiatrimottagning 1 Lund
Sofiavägen 2 E, 221 85 Lund
per.andren@med.lu.se
+46 70-435 17 40

Dr Matti Cervin
Lund University
Department of Clinical Sciences
Barn- och ungdomspsykiatrimottagning 1 Lund
Sofiavägen 2 E, 221 85 Lund
matti.cervin@med.lu.se

+46 73-719 97 00

2 SUMMARY

**Title:**	Clinical efficacy and cost-effectiveness of Internet-delivered cognitive-behaviour therapy for children and adolescents with body dysmorphic disorder: a randomised controlled trial
**Short title:**	Internet-delivered cognitive-behaviour therapy for child and adolescent body dysmorphic disorder
**Objectives:**	The primary objective is to determine the clinical efficacy of a therapist-guided, Internet-delivered cognitive-behaviour therapy (ICBT) programme for body dysmorphic disorder (BDD) in reducing BDD symptom severity in children and adolescents with BDD versus an active comparator consisting of a therapist-guided, Internet-delivered relaxation treatment (IRT) for BDD.<br><br>The secondary objectives are to establish the 6-month durability of the treatment effects and to assess the cost-effectiveness of ICBT, compared with IRT, from multiple perspectives.
**Type of trial:**	Multisite parallel-group randomised controlled superiority trial.
**Rationale for the study:**	BDD is a prevalent and impairing disorder that tends to have a chronic course if left untreated. Adolescent-onset BDD is associated with more severe symptoms, greater lifetime comorbidity, and higher rates of attempted suicide compared to adult-onset BDD. Therefore, early intervention is crucial. BDD can be effectively treated with cognitive-behaviour therapy (CBT), although the current evidence is rather weak and more evidence is needed. Furthermore, CBT for BDD is a highly specialised treatment and many young people do not have access to it. ICBT can be a way to increase the availability of an effective, evidence-based treatment for children and adolescents with BDD. To date, no large scale randomised controlled trial (RCT) has evaluated the clinical efficacy of ICBT for child and adolescent BDD, but the results from studies in adults with BDD treated with ICBT are encouraging. Hopefully, this study will contribute to strengthening the evidence base for the treatment of BDD in children and adolescents.
**Trial design and methods:**	Participants will be recruited nationally across Sweden and will be offered 12 modules of therapist-guided ICBT or 12 modules of therapist-guided IRT delivered over 12 weeks. Under certain circumstances, such as illness or holidays, the design allows participants to pause their therapist-support for a maximum of two weeks, which may extend the treatment

	length to a maximum of 14 weeks. All potential participants will be initially screened via the telephone or at one of the three participating sites. This will be followed by an inclusion/baseline assessment conducted either at one of the three clinics (BUP OCD och relaterade tillstånd [Region Stockholm], BUP Specialmottagning [Västra Götalandsregionen] or Barn- och ungdomspsykiatrimottagning digital behandling Skåne [Region Skåne]) or, if face-to-face assessments are not feasible, via a secure video application. Participants who are eligible and have consented will be randomised to one of two trial arms. Participants in the comparator group (IRT) will be offered to cross-over to the ICBT intervention after the primary endpoint.<br><br>Participants will complete outcome measures at baseline, post-treatment, and 1- (primary endpoint), 3-, and 6-months post-treatment. Follow-up assessments will be conducted at the clinic or via a secure video application, in both cases complemented with online self- and caregiver/parent-reported questionnaires.<br><br>The primary outcome variable is BDD symptom severity measured by the Yale-Brown Obsessive Compulsive Scale modified for BDD, Adolescent version (BDD-YBOCS-A) at the primary endpoint (1-month follow-up post-treatment). Based on BDD-YBOCS-A scores, responder and remission rates at all follow-up points will be calculated. Response will be defined as ≥30% reduction from baseline; full or partial remission will be defined as a score ≤16.<br><br>Secondary outcomes will include clinician-reported measures of global functioning (Clinical Global Impairment-Severity and Improvement; Clinical Global Assessment Scale), as well as self- and caregiver/parent-reported, for example: the Appearance Anxiety Inventory to assess cognitive and behavioural aspects related to BDD (self-reported), the Short Mood and Feelings Questionnaire (self- and caregiver/parent-reported versions) to assess depressive symptoms, the Work and Social Adjustment Scale – Youth/Parent version to assess functional impairment, the CRAFFT to assess alcohol and substance use, and measures of quality of life, adverse events, treatment credibility, and satisfaction
**Participant time in trial:**	Approximately 10 months (or 15 months for those who will cross-over).
**Total trial duration:**	Approximately 38 months (from first participant enrolment to last participant's final follow-up assessment, including those who will cross-over).
**Planned trial sites:**	The study will be coordinated from the Department of Clinical Neuroscience at Karolinska Institutet (the Sponsor).

	Collaboration agreements will be established with each of the 3 clinic sites: BUP OCD och relaterade tillstånd (Region Stockholm), BUP Specialmottagning (Västra Götalandsregionen), and Barn- och ungdomspsykiatrimottagning digital behandling Skåne (Region Skåne). Each of the three sites will assess and treat participants from their own region, and occasionally from adjacent regions.
**Sample:**	A total of 154 children and adolescents diagnosed with BDD and their primary caregivers.
**Brief eligibility criteria:**	Eligible participants will be aged 12-17 years and have a DSM-5 diagnosis of BDD with a total BDD severity score on the BDD-YBOCS-A ≥24. Main exclusion criteria include having received CBT for BDD within the 12 months prior to enrolment; dosage change (or introduction/cessation) of serotonin selective reuptake inhibitor medication within the 6 weeks prior to inclusion; a history of organic brain disorder, intellectual disability, psychosis, bipolar disorder, eating disorder, severe depression or alcohol/substance dependence; immediate risk to self or others; or a documented suicide attempt in the last 12 months. Full details are presented in section 5.4.
**Statistical methodology and analysis:**	Data will be analysed using a pre-specified intention-to-treat statistical analysis plan. The primary outcome will be analysed using a linear mixed-effects model. Secondary outcomes will be analysed using analogous methods. We will also conduct a health economic evaluation of the intervention from a health organization payer, healthcare resource use, and societal perspective.

3 INTRODUCTION

Body dysmorphic disorder (BDD) is a psychiatric condition characterized by a preoccupation with perceived defects or flaws in physical appearance, leading to time-consuming repetitive behaviours (e.g., mirror checking, camouflaging), mental acts (i.e., comparing), and avoidance (e.g., social situations). BDD is a chronic disorder which persists for many years if left untreated (1) and causes significant distress and impairment (2). BDD is associated with social withdrawal, reduced academic performance, and school dropout (3,4). The prevalence of BDD is approximately 2% in community samples of both adolescents and adults (1,5,6). BDD usually emerges during adolescence, with a reported mean age of onset between 12-16 years (3,7,8). Retrospective studies show that early age of onset is associated with greater illness severity and greater comorbidity with other disorders (7), indicating the importance of early detection and treatment. Nonetheless, the disorder often goes undetected in adolescents due to BDD symptoms at that age being interpreted as normal developmental concerns (4,9). Adolescents with BDD are characterized by low disorder insight, significant functional impairment, high rates of psychiatric comorbidity, suicidal ideation, and self-harm (3,4,8,10).

Cognitive-behaviour therapy (CBT) adapted for BDD is recommended in Swedish and international guidelines (11–13). The evidence base stems from a small number of randomized controlled trials (RCTs) in adults (samples ranging from 19-120 individuals) (14,15). By contrast, there is only one small pilot RCT in children and adolescents, conducted in the UK (4). In that study, participants in the CBT condition (n=15) experienced a significant reduction of BDD symptoms, compared to a control psychoeducation condition (n=15), both immediately after CBT (between-group d=1.13; 95% CI, 0.31-1.96) and two months after treatment (between-group d=0.85; 95% CI, 0.02-1.69). At the post-treatment follow-up, 35% of the participants receiving CBT were classified as responders and 19% as remitters. The gains were maintained up to 12 months after the end of the intervention in a naturalistic follow-up, where 50% of the participants receiving CBT were classified as responders and 23% as remitters (9). The treatment manual developed for that study has undergone various refinements and has been further evaluated by our research team with excellent results in a naturalistic study of 140 young people with BDD treated at specialist clinics in Stockholm, Sweden and London, England (16). This study has shown that CBT delivered in regular care by specialist teams is associated with a significant reduction of BDD symptoms (d=2.08; 95% CI, 1.81-2.35 at post-treatment and d=2.39; 95% CI, 2.05-2.72 at the 12-month follow-up).

Despite the encouraging results from these and a handful of other, smaller open studies in paediatric BDD, (4,16–18) the evidence supporting CBT for BDD in children and adolescents is scarce, and it is currently unclear if this treatment outperforms other available psychological interventions that do not require such a high level of expertise. For example, a recent study comparing an Internet-based interpretation bias modification training protocol to progressive muscle relaxation in a sample of 50 adults with BDD found that both treatments yielded significant improvements in BDD symptoms, with no between-group differences (19). The field would greatly benefit from additional controlled trials, particularly in young people, addressing the question of treatment specificity, that is, whether CBT for BDD is superior to an active control treatment.

Furthermore, CBT for BDD is a highly specialized treatment that may be difficult to access, mainly due to lack of trained professionals and geographical barriers, given that specialist services across the country are very limited. One possible solution is to deliver a low-intensity online version of the treatment with minimal remote support from a clinician, similar to a guided self-help intervention (20). This kind of guided Internet-delivered CBT (ICBT) differs from standard telepsychiatry in that the therapist does not actively deliver the treatment content in real time. Instead, the therapist provides minimal support asynchronously via a messaging system built in the online platform. This approach has the potential to be used in a stepped-care, scalable model of health care which makes a better use of the existing resources (21,22). ICBT has shown to be cost-effective, needing less therapist time per patient (compared to traditional CBT) and has a strong evidence base for a wide range of disorders in adults, adolescents, and children (23–25).

A small literature also supports the efficacy and cost-effectiveness of ICBT for adults with BDD. A programme developed in Sweden, called BDD-NET, was successfully evaluated in a feasibility study (n=23) (26), one RCT (n=94) (14), and one open trial with global inclusion (n=32) (27), with response rates in line with traditional face-to-face CBT (14) and showing sustained treatment effects up to 2 years after treatment (28). In the United States, an app-based intervention has also been successfully evaluated in a feasibility study (n=10) (29) and an RCT (n=80) (30) for adults with BDD. The above-mentioned trial comparing an interpretation bias modification training with progressive muscle relaxation (19), also delivered either of the interventions over the Internet and showed significant improvements in both conditions. Our research team in Stockholm has recently conducted a pilot trial (n=20) testing the feasibility, acceptability, and safety of an ICBT program for young people with BDD, with promising results (ClinicalTrials.gov: NCT05078320) (31). The current trial represents an extension of this preliminary work and will be evaluating efficacy and cost-effectiveness of ICBT vs. an active comparator consisting of a relaxation treatment in a fully powered RCT.

4 OBJECTIVES

Primary:

1. To determine the clinical efficacy of therapist-guided ICBT for BDD in reducing BDD symptom severity (as measured by the Yale-Brown Obsessive Compulsive Scale modified for BDD, Adolescent version; BDD-YBOCS-A) in children and adolescents with BDD, compared with an active comparator (therapist-guided, Internet-delivered relaxation treatment; IRT) for BDD. The primary endpoint is the 1-month follow-up post-treatment. We hypothesise that ICBT will be superior to IRT in reducing BDD symptom severity at the primary endpoint in children and adolescents with BDD.

Secondary:

2. To establish the 6-month durability of the treatment effects in a naturalistic follow-up. We hypothesise that participants receiving ICBT will maintain their treatment gains at the 6-month follow-up.

3. To conduct a health-economic evaluation of ICBT for BDD at the primary endpoint from a health organisation payer, healthcare resource use, and societal perspective. We hypothesise that ICBT for BDD will be more cost-effective than IRT.

5 PROJECT DESCRIPTION

5.1 DESIGN

This is a multisite parallel-group randomised controlled superiority trial for children and adolescents with BDD. Participants will be randomised to 12 modules of therapist-guided ICBT for BDD or 12 modules of an active comparator, consisting of therapist-guided IRT. The comparator aims to account for similar online access to basic information on BDD, online therapist support, and homework assignments.

The default treatment length is 12 weeks, but under certain circumstances (e.g., illness or holidays), the design allows participants to pause their therapist-support for a maximum of two weeks, which may extend the treatment length to a maximum of 14 weeks.

Participants will be followed-up at post-treatment, and 1-, 3-, and 6-months after the end of the treatment. Up to the primary endpoint (1-month follow-up), participants are encouraged to not introduce changes in their psychiatric medication status or start alternative psychological treatment for their BDD.

At the primary endpoint, participants initially assigned to the comparator will be offered to cross-over to receive ICBT for BDD. All participants will be followed up for 6 months regardless of whether they accept to cross-over or not. Participants in either group who are still in need of further treatment after ICBT will be referred to their local services (e.g., in-person CBT at one of the three clinics or referral to other appropriate services). The 3- and the 6-month follow-ups are naturalistic (i.e., non-controlled) follow-ups. Participants may be using alternative treatments in accordance with standard practice recommended by their treating clinician. Assessors and other study personnel (see section 6.2) will be blinded to treatment allocation at all assessment points. A flow chart of the trial design is shown in **Figure 1**.

Figure 1. CONSORT diagram of study design and flow of participants.

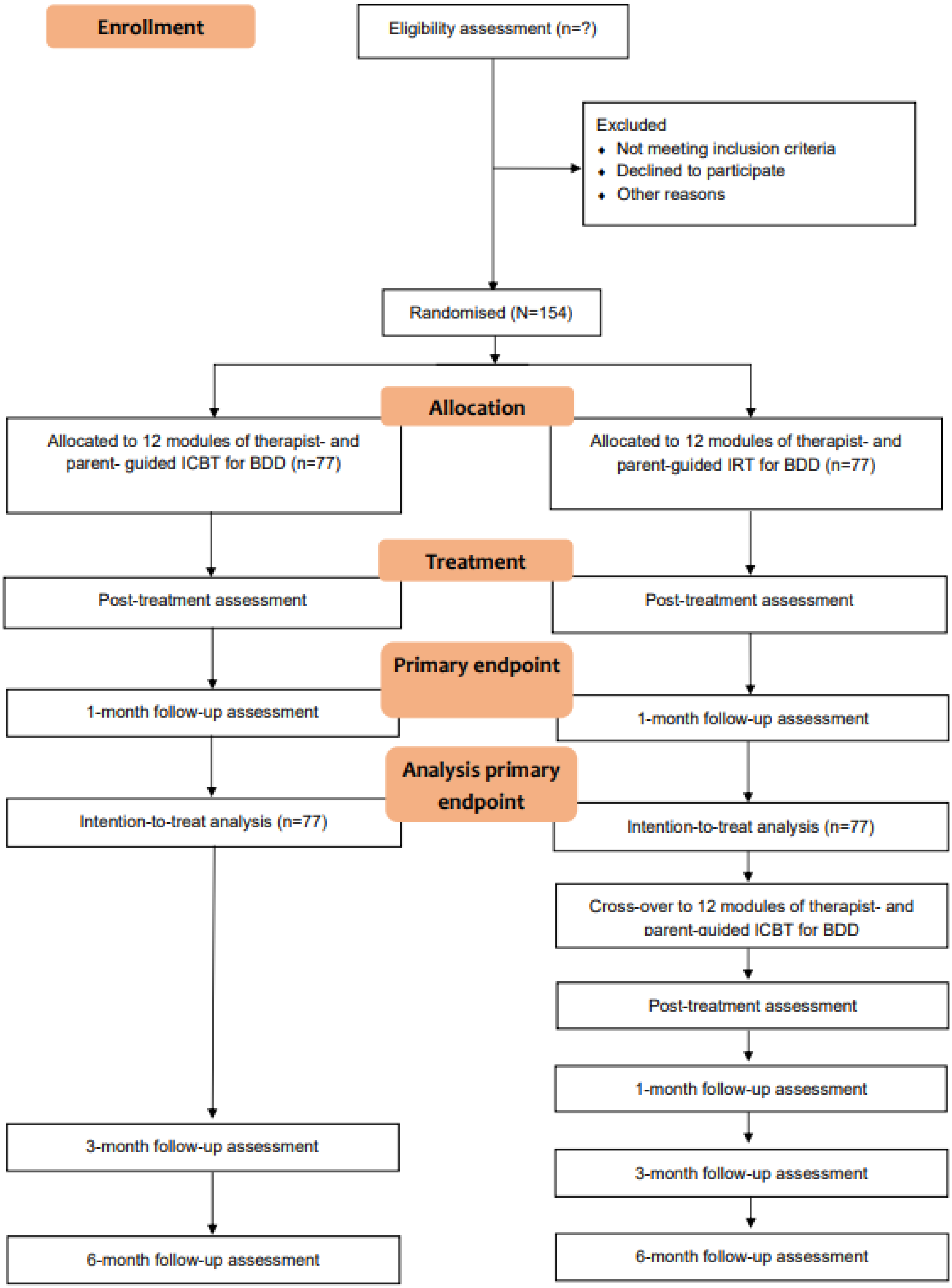

Note: BDD, body dysmorphic disorder; ICBT, Internet-delivered cognitive behaviour therapy; IRT, Internet-delivered relaxation treatment.

5.2 STUDY SITES

The study is a collaboration between the Department of Clinical Neuroscience at Karolinska Institutet (the Sponsor) and Child and Adolescent Mental Health Services (Barn- och ungdomspsykiatrin; BUP) in Region Stockholm, Västra Götalandsregionen, and Region Skåne.

The trial will be coordinated from the Department of Clinical Neuroscience at Karolinska Institutet. Assessments and delivery of treatment will be conducted at all three study sites: BUP OCD och relaterade tillstånd (Region Stockholm), BUP Specialmottagning (Västra Götalandsregionen), and Barn- och ungdomspsykiatrimottagning digital behandling Skåne (Region Skåne). Each of the clinics will accept referrals from their own regions but also from adjacent regions, according to the right to treatment principle (in Swedish, *fritt vårdval*). In these cases, the referring regions will not need to pay for the treatment. Participants will formally be considered patients at one of the three clinics while they are part of the trial, from enrolment to the 6-month follow-up.

5.3 POWER ANALYSIS

Between-group effect sizes in an RCT comparing face-to-face CBT with an active comparator in youth with BDD (4) and an RCT comparing ICBT with an active comparator in adults with BDD (14) were *d*=1.13 and *d*=0.94, respectively. Other ICBT trials in the paediatric population similar to our proposed study have reported smaller effect sizes. For example, an ICBT study of adolescent obsessive-compulsive disorder (OCD) (32) and an ICBT study of adolescent social anxiety disorder (33) reported between-group effect sizes of *d*=0.69 and *d*=0.64, respectively. Similarly, an adult BDD trial with an active control reported an effect size of *d*=0.76 (15). Thus, we have initially powered our study to be able to detect at least a moderate effect size (e.g., *d*=0.50). A sample size of 128 participants was calculated to be sufficient with a power of 80%, with a 2-tailed α=0.05. An additional 20% (15) of participants will be added to account for potential dropouts. Hence, we aim to recruit a total of 154 participants (77 per group).

5.4 INCLUSION AND EXCLUSION CRITERIA

5.4.1 INCLUSION CRITERIA

1) A primary diagnosis of BDD, based on the diagnostic criteria of the 5th edition of the Diagnostic and Statistical Manual of Mental Disorders (2).
    - Confirmed by the assessor at the inclusion assessment using a semi-structured diagnostic interview. In cases where it is challenging to rule out the presence of actual physical defects that are clearly noticeable (e.g., if the area of concern is the genitalia), we will make a referral to an appropriate service for an expert opinion.
2) A total BDD symptom severity score on the BDD-YBOCS-A ≥24.
    - Confirmed by the assessor at the inclusion assessment.

3) Age between 12 and 17 years.
    - Confirmed by the caregiver/parent and subsequently by the medical record.
4) A minimum of one available caregiver/parent being able to co-participate and support the child/adolescent throughout the treatment.
    - Confirmed by the caregiver/parent at the inclusion assessment.
5) Regular access to a computer connected to the Internet and a mobile phone to receive SMS messages (one of each per family is enough).
    - Confirmed by the caregiver/parent at the inclusion assessment.

5.4.2 EXCLUSION CRITERIA

1) Previous CBT for BDD for a minimum of five sessions with a qualified therapist within the 12 months prior to the inclusion assessment.
    - Confirmed by the caregiver/parent at the telephone screening and/or the inclusion assessment and by the medical record.
2) Simultaneous psychological treatment for BDD or for any psychiatric comorbidity.
    - Confirmed by the caregiver/parent at the telephone screening and/or the inclusion assessment and by the medical record.
3) Initiation, change of dosage or cessation of any selective serotonin reuptake inhibitors (SSRI) or antipsychotic drugs within the six weeks prior to the inclusion assessment.
    - Confirmed by the caregiver/parent at the telephone screening and/or the inclusion assessment and by the medical record.
4) A diagnosis of organic brain disorder, intellectual disability, psychosis, bipolar disorder, eating disorder, severe depression or alcohol/substance dependence.
    - Confirmed by the caregiver/parent at the telephone screening and/or the inclusion assessment, with supplemental information from the semi-structured diagnostic interview and by the medical record.
5) Immediate risk to self or others requiring urgent medical attention or inpatient care, such as suicidality, or repeated self-injurious behaviours.
    - Confirmed by the caregiver/parent at the telephone screening and/or the inclusion assessment.
6) A documented suicide attempt in the last 12 months.
    - Confirmed by the caregiver/parent at the telephone screening and/or the inclusion assessment or by documentation in the medical record.
7) Child/adolescent and caregiver/parent not able to read and communicate in Swedish.

- Confirmed by the caregiver/parent at the telephone screening and/or the inclusion assessment.

8) Having a close relationship to an already included participant (e.g., sibling, cousin), to avoid being randomised into two different arms, with the risk of information "leaking" between the groups.
    - Confirmed by the caregiver/parent or assessor at the telephone screening and/or at the face-to-face or video conference inclusion assessment.

5.5 MEASURES

Assessments will be conducted at baseline, mid-treatment, post-treatment, and 1-, 3- and 6-month post-treatment face-to-face at the clinic or via videoconference software. If technical problems with the videoconference software arise, the assessment will be made over the telephone instead (with the family on loudspeaker). The assessments will be conducted with both the child/adolescent and at least one of the caregivers/parents, but the assessment can also be conducted with only the participant or the caregiver/parent if needed (this will be registered).

Clinician-rated/administered measures will first be recorded on paper, then inputted into the trial database in the study platform, called BASS. BASS is provided by the Karolinska Institutet e-Health Core Facility. Child/adolescent- and caregiver/parent-reported measures will be completed via the Internet directly into BASS. More information about BASS can be found in section 7.1.1. For the specific time points of administration of each measure, see **Table 1**.

In order to account for delays in data collection due to personal circumstances (e.g., holidays, illness), there will be +14 days' time period for data collection during treatment and for the post-treatment and primary endpoint, and a ±1 month time period for the 3- and 6-month follow-ups. If these time windows are missed, the data point will be considered missing.

Table 1. Assessment points for each outcome.

Assessment point / Outcome	Baseline	Mid-treatment	Post-treatment	1-month follow-up (primary endpoint)	3-month follow-up	6-month follow-up
**Baseline measures**						
OCD-RD interview (clinician-administered)	X					
DIAMOND-KID (clinician-administered)	X					
**Clinician-rated outcomes**						
BDD-YBOCS-A **(primary outcome measure)**	X		X	X	X	X
Response and remission rates			X	X	X	X
CGI-S	X		X	X	X	X
CGI-I			X	X	X	X
CGAS	X		X	X	X	X
**Child/adolescent-reported outcomes**						
AAI	X		X	X	X	X
SMFQ + suicide item	X	Weekly	X	X	X	X
GAD-7	X		X	X	X	X
DSHI-Y-7	X		X	X	X	X
CRAFFT	X		X	X	X	X
WSAS-Y	X		X	X	X	X
CHU9D	X		X	X	X	X
WAI-C		After 3 weeks				
CSQ-8			X			
TCES		After 3 weeks				
PEAS/PRAS		After 4 & 12 weeks				
Treatment preference	X		X			
**Caregiver/parent-reported outcomes**						
SMFQ	X	Weekly	X	X	X	X
WSAS-P	X		X	X	X	X
TiC-P	X		X	X	X	X
WAI-P		After 3 weeks				
CSQ-8			X			
TCES		After 3 weeks				
Treatment preference	X		X			
**Other measures**						
Demographic data – clinician	X					
Demographic data – caregiver/parent-reported	X					
Areas of concern and cosmetic procedures – clinician	X		X	X	X	X
School absenteeism – clinician	X		X	X	X	X
Concurrent interventions – clinician	X		X	X	X	X
BASS platform usage data + therapist time – clinician			X			

Completed modules – clinician			X			
Adverse events questionnaire – child/adolescent and caregiver/parent reported		After 6 weeks	X	X	X	X

Note: *OCD-RD interview*, Obsessive-compulsive disorder and related disorders interview; *DIAMOND-KID,* Diagnostic Interview for Anxiety, Mood, and OCD and Related Neuropsychiatric Disorders - the Child and Adolescent Version; *BDD-YBOCS-A,* Yale-Brown Obsessive Compulsive Scale Modified for Body Dysmorphic Disorder, Adolescent version; *CGI-S,* Clinical Global Impression Scale – Severity; *CGI-I,* Clinical Global Impression Scale – Improvement; *CGAS,* Children's Global Assessment Scale; *AAI,* Appearance Anxiety Index; *SMFQ,* Short Mood and Feeling Questionnaire; GAD-7 Generalized Anxiety Disorder – 7 item scale; *DSHI,* Deliberate Self-Harm Inventory Youth version; *CRAFFT,* acronym for the key words Car, Relax, Alone, Forget, Friends and Trouble; *WSAS-Y/P,* Work and Social Adjustment Scale – youth and parent version; *CHU9D,* Child Health Utility 9D; *WAI-C/P,* Working Alliance Inventory – child and parent version; *CSQ-8,* Client Satisfaction Questionnaire; *TCES,* Treatment Credibility and Expectancy Scale; *PEAS,* Patient Exposure Adherence Scale; *PRAS,* Patient Relaxation Adherence Scale; *TiC-P,* Trimbos/iMTA Questionnaire for Costs associated with Psychiatric Illness.

5.5.1 BASELINE MEASURES

Diagnosis of BDD and comorbid psychiatric disorders

Psychiatric diagnoses at baseline will be based on Diagnostic and Statistical Manual of Mental Disorders (DSM) diagnostic criteria. A semi-structured interview based on DSM-5 criteria, the Diagnostic Interview for Anxiety, Mood, and OCD and Related Neuropsychiatric Disorders – the Child and Adolescent Version (DIAMOND-KID) (34), will be used. Additional obsessive-compulsive related comorbidities not included in the DIAMOND-KID (e.g., olfactory reference syndrome) will be assessed using separate modules from the Structured Clinical Interview for DSM-IV (SCID-I) (35).

5.5.2 PRIMARY OUTCOME

Yale-Brown Obsessive Compulsive Scale Modified for Body Dysmorphic Disorder, Adolescent version (BDD-YBOCS-A)

The main clinical outcome measure is the clinician-rated BDD-YBOCS-A (36), measured post treatment, 1- (primary end-point), 3-, and 6-months after treatment completion. The BDD-YBOCS-A contains twelve items ranging from 0 to 4. It includes five questions on obsessions, five on compulsions, one about insight, and one to measure behavioural avoidance. The total BDD severity score ranges from 0 to 48. This semi-structured interview has good psychometric properties (37) and is the gold standard in the BDD field to assess symptom severity, which will facilitate comparability with previous BDD trials. The BDD-YBOCS-A takes between 15 to 45 minutes to administer (generally taking longer time at baseline than at the subsequent follow-ups). When possible, the post- and follow-up assessments will be conducted by the same blinded assessor (for the same participant).

All baseline assessors and blinded raters of the BDD-YBOCS-A will be extensively trained before the start of the trial. The training will be supervised by clinical experts (DR, AB).

Extensive measures will be implemented to ensure blinding integrity. Training procedures are the following:

1) Training will consist of completing the BDD assessment module in an Internet-based, interactive training course in paediatric BDD for clinicians. The course consists of 2 Internet-modules (about 3 hours) that focus on BDD, such as psychoeducation, clinical characteristics, screening and assessment with BDD-YBOCS-A, and differential diagnosis. The course is delivered online and includes texts, videos, and exercises. During the training, raters will have the possibility to ask questions and discuss with the clinical experts via the Internet platform.
2) At the end of the course, raters will have to complete a test where their knowledge on assessing BDD as well as their skills in assessment and differential diagnosis will be evaluated. A part of the test will be a pre-recorded video of a BDD-YBOCS-A assessment which will be scored by the raters. Experienced raters (i.e., those working at an OCD and related disorders specialist clinic and have worked with BDD patients for at least a year, or have assessed at least three patients with BDD) will have to rate three pre-recorded BDD-YBOCS-A audio files. Raters with less experience will have to rate six pre-recorded BDD-YBOCS-A audio files. Raters scores will be compared to those of an expert rater to determine the extent of the agreement. The raters will have to be within a margin of +/- 2 on the BDD-YBOCS-A total score, compared to the expert rater score.
3) Raters who do not meet the criteria will be given additional training and asked to score additional audio-recorded BDD-YBOCS-A assessments until the specified agreement criteria are met.
4) The raters will also read a short instructional text describing how the BDD-YBOCS-A is used within this trial and they will be given information about study routines and the trial database.
5) Once a semester raters will listen to audio recordings of BDD-YBOCS-A assessments together, rate them individually, and discuss their ratings. These recordings will be used to measure inter-rater reliability throughout the trial. Raters deviating from the margin of +/-2 point will be given additional training and asked to score new BDD-YBOCS-A assessments until the specified agreement criteria are met.

5.5.3 SECONDARY CLINICIAN-RATED OUTCOMES

Response and remission rates

Treatment response and full or partial remission rates will be calculated at each follow-up point. Response will be defined as a reduction ≥30% on the BDD-YBOCS-A from baseline. Full or partial remission will be defined as a total score ≤16 on the BDD-YBOCS-A (38).

Clinical Global Impression Scale – Severity (CGI-S) and Improvement (CGI-I)
The CGI-S and CGI-I (39) are clinician-rated single item scales of symptom severity and

symptom improvement, respectively, in this case in reference to the BDD symptoms. The CGI-S is used as an overall rating of BDD severity, and the rating is made on a seven-point scale from 1 ("normal, not at all ill") to 7 ("among the most extremely ill patients"). The CGI-I is used to assess the level of improvement, always compared to baseline, and it is rated on a seven-point scale from 1 ("very much improved") to 7 ("very much worse"). All assessors will be trained in the use of the CGI-S and CGI-I and will rate them in addition to the BDD-YBOCS-A (see above). The assessors will also practise rating the CGI-S and CGI-I together during the regular training. The CGI-S and CGI-I can be completed in less than a minute by an experienced rater.

Children's Global Assessment Scale (CGAS)

The CGAS (40) is a clinician-rated single item scale of the global functioning of a young person during the last month (not restricted to the BDD symptoms only). It integrates psychological, social, and academic functioning as a measure of psychiatric disturbance. The questionnaire has established validity and reliability (40). All assessors will be trained in the use of the CGAS and will rate them in addition to the BDD-YBOCS-A (see above) together during the regular training. The CGAS can be completed in less than a minute.

5.5.4 SECONDARY CHILD/ADOLESCENT AND CAREGIVER/PARENT-REPORTED OUTCOMES

The full battery of self-reported measures will take about 40-45 minutes to complete for the child/adolescent and 35-40 minutes for the caregiver/parent.

Appearance Anxiety Index (AAI)

The Appearance Anxiety Inventory (AAI) is a self-reported measure that covers cognitions and behaviours typical of BDD (41). It consists of 10 items, with a total score ranging from 0 to 40, and it includes two subscales: avoidance and threat monitoring. The questionnaire has shown good psychometric properties (41). The AAI is only filled out by the child/adolescent.

Short Mood and Feeling Questionnaire – child version and parent version (SMFQ) + additional suicide item (only child/adolescent)

The SMFQ (42) is a 13-item self-reported measure of depressive symptoms. Each item is scored on a 3-point scale. The total score is derived by adding up the values for the 13 items. The questionnaire has established validity and reliability (42).

Additionally, for this trial, we have added an item aimed at assessing suicide risk where the participants are asked to choose one out of four alternatives that best describe how they have felt during the past week: 1. I don't think about death or about killing myself; 2. I think about death but not about killing myself; 3. I think about death and about killing myself, but I would never do it; 4. I think about death and I am thinking about killing myself. The parent version does not include the additional suicide item.

Generalized Anxiety Disorder – 7 item scale (GAD-7)

The GAD-7 (43) is a measure of anxiety rated by the child/adolescent. It consists of 7 items and each item is rated from 0=not at all to 3=almost every day, yielding a total score of 0-21.

Deliberate Self-Harm Inventory – Youth Version (DSHI-Y-7)

The DSHI-Y-7 is a 7-item self-reported measure to assess the presence and frequency of participants' non-suicidal self-injury. It will help the investigators to assess risk behaviours throughout the trial. The DSHI-Y-7 is a reduced version of the adolescent version of the DSHI-17 (44). The DSHI-17 is an empirically supported measure of various aspects of non-suicidal self-injury in adolescents and has demonstrated good test-retest reliability and adequate concurrent validity among adolescents (44). The reduced version of the questionnaire used in the trial has been adapted by colleagues of the research team and used in previous investigations in the adolescent population. At baseline, the complete DSHI-Y-7 will be administered. At the post-treatment and the follow-up assessments, we will not ask the questions about self-harm during the last 12 months as this information would have been covered in the previous assessments. The DSHI-Y-7 is only filled out by the child/adolescent.

CRAFFT

The CRAFFT (acronym for the key words Car, Relax, Alone, Forget, Friends and Trouble) is a short self-reported instrument designed to identify substance use and substance use disorder (including nicotine, alcohol, and other drugs) (45). It consists of ten yes or no questions and has shown good psychometric properties (46).

Work and Social Adjustment Scale – youth (WSAS-Y) and parent version (WSAS-P)

The Work and Social Adjustment Scale – Youth and Parent Versions (WSAS-Y and WSAS-P, respectively) are short, self- and caregiver/parent-reported instruments assessing functional impairment in five areas: school, daily situations, social activities, leisure activities, and relationships (47). The WSAS-Y/P are based on the original WSAS for adults (48). Both versions have excellent psychometric properties (47).

Child Health Utility 9D (CHU9D)

The CHU9D (49) is a child/adolescent-rated 9-item self-reported generic preference-based measure of health-related quality of life designed specifically for use in economic evaluation targeting children and adolescents. Each item is scored from 1 to 5, yielding a total score ranging from 9 to 45. These scores are used to estimate utility value weights using a scoring algorithm. Utility weights range between zero (denoting the worst possible health state; i.e., death) and one (denoting the best possible health state). The questionnaire has been validated on a Swedish sample and has established validity and reliability (49). The CHU9D will be used to estimate quality-adjusted life years (QALYs).

Trimbos/iMTA Questionnaire for Costs associated with Psychiatric Illness (TiC-P)

The TiC-P (50) will be used to assess healthcare and societal resource use. The questionnaire includes items on healthcare resource use (e.g., healthcare visits), supportive resources (e.g., private tutoring), medications, prescription-free drugs, school absenteeism, academic productivity loss, and parental productivity loss and is frequently used in health economic studies. The TiC-P version used in the study has been adapted by the research team for the use among young people and caregivers/parents. The timeframe captured with the TiC-P will be adapted for each assessment point in order to captures the last month at baseline, the last three months at post, the last month at the 1-month follow-up, the last two months at the 3-month follow-up and the last three months at the 6-month follow-up. The instrument is answered by the caregiver/parent.

Working Alliance Inventory – child (WAI-C) and parent version (WAI-P)

The WAI (51) is a 6-item scale measuring the participants and the caregivers/parents perceived working alliance with their therapist. The scale is rated from 1 to 7, yielding a total score of 7-49. The WAI is used to control for non-specific therapeutic effects between the different treatment conditions.

Client Satisfaction Questionnaire (CSQ-8)

To assess treatment satisfaction, in both the child/adolescent and their caregivers/parents, we will use the CSQ-8 (52), which has 8 items rated from 1 to 4, yielding a total score of 9-36. The scale measures different aspects of satisfaction with treatment such as perception of treatment and if the treatment adequately addressed the participants needs and overall satisfaction. The CSQ-8 has a high internal consistency and correlates with therapists' estimates of client satisfaction (52).

Treatment Credibility and Expectancy Scale (TCES)

The TCES (40) will be administered to measure treatment credibility or expectancy of treatment outcome. The TCES includes 5 items rated from 0 to 10, yielding a total score of 0-40, where higher scores indicate higher credibility. The TCES will be administered to both the child/adolescent and the caregiver/parent.

Patient Exposure Adherence Scale (PEAS) and Patient Relaxation Adherence Scale (PRAS)

The PEAS (53) is a 3-item measure of patient adherence to exposure and response prevention with 3 items rated from 0 to 6, yielding a total score of 0-18. For the current trial, the measure will be administered as a self-rated version in the ICBT group. A corresponding version (Patient Relaxation Adherence Scale; PRAS) has been developed by the research team and will be administered to the participants in the comparator group to assess adherence to the relaxation treatment. The PEAS/PRAS is answered by the child/adolescent.

Treatment preference

Two items will be administered to the caregiver and to the child/adolescent to assess treatment format preference (i.e., if the participant would have preferred to receive Internet-delivered treatment or in-person treatment for his/her symptoms or if it does not matter), as well as how important the participant believe it is to be able to choose which treatment format he/she receives (rated on a 5-point scale). The questionnaire has been developed by the research team.

5.5.5 OTHER MEASURES

Demographic data – clinician

Following the face-to-face or video conference inclusion assessment, the assessor enters baseline information into the trial database. This includes information such as screening number, age, sex, primary responsible caregiver assisting during the treatment, age of BDD onset, self-reported comorbid diagnoses, history of suicide attempts, family history of OCD and related disorders (including BDD), family history of other psychiatric disorders, current medication for BDD or other psychiatric disorders, previous psychological treatment for BDD, and distance to the clinic (in kilometers).

Demographic data – parent-reported

At baseline, the caregiver/parent will answer an online questionnaire designed by the research team asking about the caregiver's relation to the child/adolescent, who the child/adolescent lives with, if the child/adolescent has any siblings, the caregivers' educational level, the caregivers' occupation, where the child/adolescent and caregivers were born, how the caregiver learned about the trial, and if the child/adolescent has been in previous contact with health care services due to her/his BDD or other psychiatric symptoms.

Areas of concern and cosmetic procedures – clinician

Clinicians will also ask participants about their main body areas of concern, using a standardized checklist of 28 different parts of the body. Additionally, clinicians will ask about current desire for and/or whether the patient had undergone any past cosmetic/surgical procedure related to their BDD concerns.

School absenteeism – clinician

The assessor will evaluate school absenteeism during the past month at all assessment points. The assessor will ask the participant and the caregiver if the participant has full, partial or no school attendance (i.e., school drop-out). If they have partial school attendance (e.g., missing school days and/or being late to school frequently), the participant/caregiver will be asked to specify how many full days during the past month the participant has missed and how many days they have been late and/or missed part of a school day (specified as more than one hour

per school day). In case of partial or no attendance, the assessor will ask if the reason is entirely, partially or not at all related to the BDD symptoms.

Concurrent interventions – clinician

To assess what other treatments the child/adolescent is accessing during the trial period, the family is interviewed by the assessor at post-treatment and subsequent follow-ups. This includes questions about medications (type, dose, indication, and time period) and psychological treatment (type, number of sessions, indication, and time period).

BASS platform usage data + therapist time – clinician

Usage data will be extracted from the BASS platform and entered into the trial database. These data are collected for each participant, including number of completed modules, number of logins, number of messages sent to the therapist, and the name of the therapist.

Telephone is not the primary way of contact during the treatment, but when used, each therapist will log the time in an Excel spreadsheet. Both therapist time in the treatment platform (automatically recorded within the system) and telephone time will be subsequently (at post-treatment) entered into the trial database as 'therapist time'.

Completed modules – clinician

The concept of "treatment completion" is not straightforward in ICBT trials, as the number of completed modules does not necessarily reflect the participant's engagement with the treatment. For this reason, several studies find a poor correlation between the number of completed ICBT modules or chapters and treatment outcomes (54). In this trial, and for both treatment conditions, the first four modules contain the minimum amount of information required for the participants to understand the treatment rationale and the tasks that they are supposed to carry out. We will report descriptive statistics on the proportion of individuals in each group having completed at least four modules.

Adverse events

A questionnaire will be administered to systematically assess adverse events at mid-treatment, post-treatment, 1-month, 3-month, and 6-month follow-up. The questionnaire is developed by the research team and includes a checklist with expected adverse events based on previous trials as well as a free text box where other, not listed adverse events can be registered. See section 9 for more information.

6 PROCEDURE

6.1 RECRUITMENT

6.1.1 RECRUITMENT RATE

Recruitment is planned to be conducted throughout a 24-month period. Our sample size calculations show we need to recruit 154 participants. The 24-month period is in practice a 19-month period, if subtracting 2.5 months each year when potential participating families are on holidays (8 weeks during summer and 2 weeks around Christmas). During these 19 months, the average recruitment rate needs to be approximately 8 participants per month (about 2 participants/week). As there is often a lag period at the beginning of recruitment, before our national advertising campaign takes full effect, we anticipate a slightly slower start to recruitment.

6.1.2 SCREENING AND RECRUITMENT PROCEDURES

We plan to mainly recruit through clinician-referrals to our three clinics BUP OCD and relaterade tillstånd (Region Stockholm), BUP Specialmottagning (Västra Götalandsregionen), and Barn- och ungdomspsykiatrimottagning digital behandling Skåne (Region Skåne). All participants who are referred to either of the three clinics will, if eligible, be offered participation in the trial. We will accept self-referrals from all over Sweden, and plan to advertise the trial to clinics across Sweden, patient organisations, as well as directly to the public via our website and social media. If needed, we may also publish paid advertisements in traditional media. This broad recruitment strategy will ensure that the results are more generalizable to the entire BDD population of Sweden.

All potential participants will initially be contacted via the telephone by a member of the research team, or in some cases approached face-to-face at the clinics for an initial screening. The main purpose of the preliminary screening is to briefly inform about the trial, perform a preliminary screening of eligibility (see section 5.4 for details), and answer any questions that the families may have. By default, the initial screening will be done with the caregiver/parent, but the child/adolescent can in some cases provide additional information.

If preliminarily eligible and interested, the participants will then be booked for an inclusion assessment, either face-to-face at the clinic or via video (if face-to-face is not feasible). Before the assessment, the potential participants will be provided with information (via regular mail, or handed out at the clinic), including the informed consent form, an age-appropriate participant information sheet, and login information to answer baseline child/adolescent- and caregiver/parent-reported online questionnaires. This will provide the research team with important information already before the inclusion assessment, giving the opportunity to further assess potential risks (for instance high scores on the depression measures), to promote participant safety. It will also give the participants an opportunity to experience the data collection workload of the trial, making it possible for them to decline their participation at an early stage.

The participants will be encouraged to sign the informed consent form prior to (or in the very beginning of) the inclusion assessment, which will be verified by the assessor. If the inclusion assessment is conducted remotely via videoconference, the signed consent form must reach the research team (generally via mail) before the participant can be included and randomised. The consent form must be signed by the participating child/adolescent and *all* legal guardians. If both the participant and the legal guardians have BankID, the family will have the possibility to sign the consent form digitally in BASS. Otherwise, the family will sign on a paper version of the consent form. The information sheet contains information about the trial and explains that participants will become patients at one of the respective clinics and that information from their medical records will be accessed as part of the study. It also informs the families that the BDD-YBOCS-A interviews will be audio recorded and stored to evaluate the reliability of the ratings, and that the pseudonymised trial data may be shared with other research groups to be used in other studies, such as individual participant data meta-analyses.

The inclusion assessments will normally be conducted in person by specialist clinicians working at one of the three recruitment sites. In some cases where in person assessments are not feasible, the inclusion assessments may be conducted remotely via videoconferencing. Each of the clinics has a multidisciplinary team of clinical psychologists, child psychiatrists, and nurses. For a diagnosis of BDD, it is necessary to rule out the presence of actual physical defects that are clearly noticeable (e.g., cleft palate, supernumerary fingers). If this is not possible or appropriate during the initial assessment, either at the clinic or remotely (e.g., genitalia need to be explored), we will make a referral to an appropriate service for an expert opinion.

The assessor will explain the aim, methods, benefits, and hazards of participating. Further, she/he will explain that the participants are under no obligation to enter the trial and that they can withdraw at any time, without having to give a reason. The assessor will then gather socio-demographic information and administer the OCD-RD interview, DIAMOND-KID, and BDD-YBOCS-A.

Before inclusion, each assessor must discuss the potential participant to include with the corresponding local study coordinator or discuss it as a group in the local weekly meetings held at the corresponding study site. If needed, the local study coordinator can also discuss the case further with the central study coordinator. If all inclusion criteria, and no exclusion criteria, are met, the participant will be included in the trial. No deviations or waivers to the eligibility criteria are allowed. If the assessor is uncertain about whether a potential participant is eligible, the Principal Investigator will be informed and have the final decision.

When the consent form has reached the research team (and is confirmed to be correctly signed), and all baseline measures have been answered, the participant will be assigned a trial ID and be randomised. The signed consent forms (for families who do not consent digitally) will be stored in a locked, fire-proof cabinet file cabinet. A log will be kept matching screening IDs with trial IDs at each trial site. The participants will be invited to choose which day to start treatment, as long as they start within one week. Even if the participant has not logged in to the platform within one week, the treatment is considered as started. In case the participant wants to start at a later day due to, for example, holidays, the BDD-YBOCS will be readministered over the telephone to verify that inclusion criterion #2 is still met.

If the participant is eventually not included in the trial (regardless of the reason), but still requires clinical attention, our team will initiate a referral to other suitable services, whenever possible.

6.2 RANDOMISATION, ENROLMENT, AND MASKING

Participants will be randomised at a 1:1 ratio to ICBT or IRT using randomly varying block sizes. Randomisation will be conducted using an online randomisation service (55), set up and monitored by the Karolinska Trial Alliance (56). One or several researchers (according to a task delegation list) will be responsible for the enrolment of participants, randomisation, and assigning participants to therapists. Participants will be informed that they will be allocated to one of two psychological treatments for BDD.

All study personnel who can be blinded to study aims/hypotheses and group allocation, will be blinded. Assessors conducting post-treatment and follow-up assessments will be external to the research team and blinded to study aims/hypotheses and treatment allocation for the full duration of the trial (up to the 6-month follow-up). The assessors will not receive any information about the study design, objectives or treatment conditions in order to maximise the blinding integrity. See **Table 2** for information about what individuals are blinded to study aims/hypotheses and/or group allocation. At each follow-up assessment, participants will be reminded by their assessor to not reveal details about their treatment.

Table 2. Individuals who are blinded to study aims or hypotheses and group allocation.

Individuals involved in the trial	Study aims/hypotheses	Group allocation
Principal Investigator	No	Yes
Co-investigators	No	Yes
Trial coordinator	No	No
Trial therapists	No	No
Study participants	Yes	No
Outcome assessors	Yes	Yes
Data managers	No	Yes
External monitors (KTA)	No	No
Statistician	Yes	Yes
Health economist	No	Yes

The primary analysis will be performed after the trial's final participant has finished his/her 1-month follow-up assessment (primary endpoint). The trial will end when the trial's final participant has finished his/her 6-month follow-up assessment (also including the subsequent

assessments, up to the 6-month follow-up, of the participants that will cross-over to receive ICBT after IRT).

When the statistical analyses are completed, and before unblinding, different versions of the abstract will be written, each reaching different conclusions (e.g., intervention A is superior to intervention B, or vice versa). After unblinding, the correct version of the abstract will be used in the final report.

In case of a medical emergency, participants will be encouraged to immediately seek appropriate health care, and to inform their therapist about this, who in turn will inform the trial coordinator. The trial coordinator will oversee following up the incident. This whole procedure can be done without unblinding the outcome assessors, so an emergency unblinding system is not required.

6.3 TREATMENT

Both treatments are Internet-delivered, using the BASS platform (see section 7.1.1), and are therapist-guided, involving both the child/adolescent and at least one caregiver/parent. The treatments consist of two separate sets of modules, one for the child/adolescent and one for the caregiver/parent, each with separate log ins. Caregivers/parents can have up to two parallel logins to the caregiver modules, if for instance they live apart.

Each intervention consists of 12 modules, delivered over a maximum period of 14 weeks. During this time, participants (both the child/adolescent and the caregiver/parent) have regular contact with a trained therapist in the platform. The therapist will provide brief asynchronous support during the treatment, which includes feedback, answering questions, and sending reminders to complete the next module, if required. The therapists will be clinically active at the three study clinics.

Communication during the treatment is possible via traditional text messages in the online platform (resembling e-mail). The therapist usually logs in daily (on workdays; at minimum every 48 hours). Therapist time will be logged automatically by BASS. There are no specified limits to the therapist time. In our pilot trial (ClinicalTrials.gov: NCT05078320; (31) the average therapist time per participant per week was about 7 minutes/week. Participants are typically in contact with their therapist several times a week. Phone calls are possible if the participant or the therapist feel that it is needed, but are generally kept to a minimum. The participants are welcome to contact their therapist at any time, who will reply during office hours.

In both interventions, the 12 modules are open at a pace of one module per week. After the treatment has finished, the child/adolescent and caregiver/parent can continue to access all treatment modules for the whole follow-up period (up to 6 months after treatment), but without therapist-support.

6.3.1 ICBT FOR CHILD AND ADOLESCENT BDD

The CBT treatment manual was initially developed in the UK for a pilot study (4) and digitalised for the current trial. The background Intellectual Property belongs to co-investigator Professor David Mataix-Cols and colleagues at the Maudsley Hospital in London.

The first part of the treatment includes psychoeducation about BDD, how avoidance and repetitive behaviours fuel and maintain the appearance concerns, and strategies to resolve possible ambivalence towards psychological treatment (as many young people prefer cosmetic or surgical procedures). The main goal of the treatment is to help the young person to stop avoiding anxiety-provoking situations (e.g., going to school or participating in other social situations) by undertaking exposure tasks and to stop doing unhelpful repetitive behaviours and rituals (e.g., excessive mirror-checking, camouflaging, excessive use of make-up), known as response prevention. Every module also contains homework tasks that are meant to be completed between modules and mainly consist of exposure and response prevention (ERP) tasks based on the young person's individual goals.

Caregivers/parents are also often involved in the patients' rituals and avoidant behaviours (known as family accommodation), which may contribute to maintain the BDD symptoms. Incorporating caregivers/parents to the treatment facilitates the modification of these unhelpful patterns. Caregivers/parents will then receive psychoeducation, information on family accommodation and how to reduce it, and strategies to be able to assist their child/adolescent in the different ERP tasks. An outline of the content of the modules is shown in **Table 3**.

6.3.2 INTERNET-DELIVERED RELAXATION TREATMENT

The active comparator condition is an adapted version of a relaxation treatment manual used in a trial for children with OCD (57). The manual is owned by Dr Jennifer Freeman and colleagues at Brown University and was digitalised for the current study.

The treatment is designed to match the above-described ICBT for BDD in all formal aspects (e.g., platform use, treatment length, therapist-support, homework assignments) except for the module content, specifically exposure and response prevention tasks.

The first part of the treatment includes psychoeducation about BDD, how anxiety is a major contributor to BDD symptoms, and that targeting and reducing stress will have a beneficial impact on those symptoms. The main goal of the treatment is to teach the young person to relax in order to cope with anxiety and appearance concerns. Further, BDD-symptoms are also stress-sensitive (i.e., they worsen in periods of stress), therefore by learning to relax, the child/adolescent will experience fewer stress-related BDD symptoms through learning anxiety management strategies.

The intervention mainly consists of different relaxation skills such as deep breathing, progressive muscle relaxation, and guided imagery relaxation. Every module also contains homework tasks that are meant to be completed between modules and mainly consist of practicing the learnt relaxation strategies in the modules.

Caregivers/parents are also involved throughout the treatment and receive psychoeducation about BDD, information on anxiety and how to reduce it, and strategies to assist their child/adolescent in the different relaxation tasks. An outline of the content of the modules is shown in **Table 3**.

Table 3. Overview of the treatment modules for both child/adolescent and caregivers/parents in each condition.

Module	ICBT	IRT
**1**	Introduction and information on BDD	Introduction and information on BDD
**2**	Learn more about BDD and set up treatment goals	Learn more about BDD
**3**	What is exposure?	What is anxiety?
**4**	Start exposure practice	Start relaxation practice by deep breathing
**5**	Focus on response prevention	Relaxation practice: Arms, legs, and back
**6**	Consequences for family and school	Relaxation practice: Head, jaw, neck, stomach, and chest
**7**	BDD thoughts and attention training	Whole body relaxation
**8**	Full response prevention	Imagery (cognitive relaxation)
**9**	Exposure and response prevention	Relaxation with and without audio file
**10**	Exposure and response prevention	Relaxation practice
**11**	Evaluate your goals, start to look forward	Start to look forward
**12**	Plan ahead	Plan ahead

6.3.3 THERAPIST TRAINING, SUPERVISION, AND MONITORING

All therapists will receive training before the trial starts. The training will be adapted to the therapist's previous experience of BDD treatment. Generally, the training will consist of the following elements:

1) Therapists will receive training on the use of the BASS platform, information on treatments via the Internet, and information on the two treatments.
2) For the ICBT training, therapists will complete an Internet-based, interactive training course for clinicians in the assessment and treatment of paediatric BDD. The course consists of 7 Internet-modules (which take about 10.5 hours to complete) that focus on BDD. The training includes psychoeducation, information about the clinical characteristics of BDD, screening, assessment with BDD-YBOCS-A, differential diagnosis, CBT for BDD, exposure and response prevention (ERP), common challenges in treatment, and relapse prevention. The course is interactive and includes texts, videos, and exercises. During the training, the therapists will have the possibility to ask

questions and discuss with the clinical experts via the Internet platform. Experienced therapists who are working at an OCD specialist clinic and have worked with BDD patients for at least a year or have treated at least three patients with BDD will not be required to complete the Internet-based training course.

3) For the IRT training, therapists will be asked to read and familiarise themselves with the treatment manual based on Freeman et. al. (57) and participate in an online lecture. The lecture will include case examples and exercises. During the training, the therapists will have the possibility to ask questions and discuss with the clinical experts. Crucially, the therapists will also be instructed not to provide any exposure and response prevention tips in their written feedback.
4) Therapists will at the end of the training course get to answer questions about the ICBT treatment and practice applied knowledge in a test. The test will include case examples and the experts (supervisors) will answer questions and provide feedback based on the case examples.

During the trial, the trial coordinator at each site will regularly log in to the system and monitor the communication between patients and therapists, and provide feedback to therapists, to ensure adherence to the treatment protocol. The communications within the treatment platform of a random 20% of the study participants will be monitored by the trial coordinator throughout the study period. This monitoring will apply to both study arms. Regular supervision of the text communication between patients and therapists is critical for quality control and adherence to protocol (e.g., to ensure that therapists delivering IRT do not inadvertently instruct participants to engage in exposure tasks, or to practise relaxation in feared situations). Protocol violations, if any, will be recorded.

Weekly meetings will be held at each trial site to discuss treatment progress for individual patients and to follow up on clinical needs and potential adverse events. Additionally, weekly meetings will be held with the local study coordinators of each site and the central study coordinator to discuss cases.

6.4 END OF TRIAL

The trial will end when the final participant (including participants who crossed over) has reached the 6-month follow-up assessment.

6.5 PARTICIPANT WITHDRAWAL FROM TRIAL

Participants will not be withdrawn from the trial if they are inactive in treatment. If the child/adolescent states that she/he does not wish to continue her/his treatment, the caregivers/parents will still be encouraged to continue working with their separate modules. In these cases, participants will still be invited to the follow-up assessments (following intention-to-treat principles). Participants will be informed that it is helpful for the trial to collect outcome measures, even if they did not complete their treatment.

Participants are free to withdraw from the trial at any point. If participants indicate to the research team/therapist that they would like to withdraw from the trial, their personal data (name, Swedish ID number, phone number, postal address, email address) will be deleted, and they will not be contacted in the future by the research team. Previously collected data up to that point will be kept and used in the data analyses. After the withdrawal, participants will be asked to provide non-obligatory feedback regarding their reason for withdrawal. This reason (if given) will be logged for CONSORT (58) reporting purposes. Once participants have withdrawn from the trial, it will not be possible for them to re-enter or resume treatment. Withdrawn participants will not be replaced.

Failure to complete outcome measures at one follow-up time point will not imply withdrawal from the trial, and the participants will still be invited to complete measures at the subsequent follow-up time points, unless they explicitly state otherwise.

6.6 DISCONTINUATION OF TRIAL

Outcome measures will not be analysed until the last participant reaches the primary endpoint and will therefore not inform decisions to stop the trial. However, serious adverse events (see section 9.1) will be reviewed and, if there is any indication that these are linked to the intervention, consideration will be given to discontinuing the trial on the advice of the Karolinska Trial Alliance and trial Sponsor.

6.7 QUALITY CONTROL

The trial will be conducted according to Good Clinical Practice (GCP) principles. Data quality and safety aspects will be regularly monitored by an independent party, the Karolinska Trial Alliance. Statistical analyses will be conducted internally by the research team, and separately by personnel at the Karolinska Institutet Biostatistics Core Facility (www.biostatcore.ki.se) (see section 8 for more information).

Weekly meetings are planned to ensure that trial procedures are being followed, including treatment fidelity and adherence to the protocol by the therapists guiding the participants' treatments.

7 DATA MANAGEMENT

All aspects of data management of the trial will comply with the General Data Protection Regulation (GDPR) and Good Clinical Practice (GCP) principles.

Notes will be made in the medical record software according to standard routines at each of the three sites.

7.1 DATA COLLECTION AND HANDLING

Data will be collected both manually (paper Case Report Forms; CRFs) and digitally (child/adolescent- and caregiver/parent-reported questionnaires completed via the Internet directly into the trial database in BASS). Some data will also be extracted digitally from the treatment platform in BASS (see section 5.5.5).

The CRFs will not bear the participant's name. Instead, the trial identification number will be used for identification. CRFs will be stored securely (separate from the code key) in a locked file cabinet at each of the three clinics. Data from the CRFs will consecutively be entered manually into BASS by a member of the research team at each of the sites. The accuracy of the data entry for the primary outcome measure (BDD-YBOCS-A) will be checked by the Karolinska Trial Alliance.

Audio recordings will be stored in a hard drive in a locked file cabinet at each of the three clinics and consecutively be transferred to a secure server (VPN) at Karolinska Institutet. The recordings will be used for spot methodology checks and future training of trial assessors.

A delegation log will identify all the personnel with responsibilities for data collection and handling, including those who have access to the trial database. After the trial is completed, data will be archived at Karolinska Institutet.

7.1.1 BASS

BASS is a digital platform provided by the eHealth Core Facility at Karolinska Institutet (https://ki.se/en/research/ehealth-core-facility). It will be used as a data collection tool, as a database for storing the data, and as a platform to deliver the treatments. The participants and their parents will login directly into BASS to both access the treatment and complete the required measures. BASS has a fine-grained privilege-system which ensures that personnel only can view information and edit settings that pertain to their role in the research project, including only viewing their relevant participants. All data sent between the platforms and the user are encrypted using a 2048-bit SSL certificate. The system is set up so that only a port 80 and 443 is visible on the Internet to the public.

The BASS system stores names of participants, telephone numbers (to send SMS messages), and e-mail addresses (to send links to child/adolescent- and caregiver/parent-rated questionnaires). The system may contain personal sensitive information, such as text messages between the therapist and child/adolescent or caregiver/parent. Logging into the BASS platform requires username, password, and a temporary code sent by text message (i.e., two-factor authentication which is required for systems storing sensitive data) for the participants, parents, clinicians working in the trial, and research personnel. Once the trial is finished, all personal information (names and telephone numbers) will be removed from BASS.

When the trial has ended, data will be extracted from BASS by the trial coordinator, then prepared/cleaned by a different member of the research team (including adding a dummy variable for the treatment allocation; e.g., A and B), and then securely transferred to the parts responsible for independent, blinded statistical analyses (see section 8).

7.1.2 VIDEOCONFERENCE SOFTWARE

For the videoconference assessments, when required, we will use software that is currently approved for regular clinical use within the three regions. At the time of writing of this protocol, the software used in Region Stockholm is Alltid Öppet, in Västra Götalandsregionen is Cerner Virtual, and in Region Skåne is Microsoft Teams. If this changes during the study, we will update our procedures and switch to the new software in question. Whenever possible, videoconference communication will be end-to-end encrypted.

8 STATISTICAL ANALYSIS

Statistical analyses relating to the clinical efficacy and 6-month durability aims will be conducted in-house by the trial coordinator at the Stockholm site (AB) and then independently replicated by personnel at the Biostatistics Core Facility at Karolinska Institutet (www.biostatcore.ki.se), who will be blinded to both aims/hypothesis and group allocation. The economic evaluation will be conducted under the supervision of co-investigator Dr Erik Andersson at Karolinska Institutet, who will also be blinded to group allocation. A full statistical analysis plan will be finalized before the final participant has reached the primary end point. In the possible case that any information regarding statistical analyses would differ from this protocol, the statistical analysis plan is considered the original source to guide analytical decisions.

8.1 PRIMARY OBJECTIVE: CLINICAL EFFICACY OF ICBT FOR BDD

Summary of baseline data

We will follow the CONSORT guidelines in reporting our data. The demographic information collected at baseline will be summarised and presented in a table, by randomisation arm. Categorical variables will be reported as counts and percentages. Continuous variables will be summarised as means and standard deviations. Following CONSORT recommendations, no statistical tests will be performed to assess baseline differences between study arms (58).

Primary outcome analysis plan

The main analyses will follow a pre-specified intention-to-treat statistical analysis plan, which will be finalised prior to unblinding and pre-registered. The primary outcome variable is the BDD-YBOCS-A total severity score at the primary endpoint (1-month follow-up). All the randomised participants will be included in a linear mixed-effect model to estimate between-group difference in means over time. The model will include fixed effects of time (baseline, post-treatment, and 1-month follow-up), treatment group (ICBT or IRT), site (Stockholm, Gothenburg or Skåne) and interaction effects of group by time, group by site, site by time, and group by site by time, as well as random intercept to account for individual differences. If the effects of site are not significant, this variable will be dropped from the model to obtain a more

parsimonious model. Linear mixed-models use all available data, can properly account for correlation between repeated measurements on the same subject, have greater flexibility to model time effects, and can handle missing data. Between-group effect sizes will be estimated using Cohen's *d*.

The procedures for handling missing data will be described in detail in the statistical analysis plan.

Analysis of secondary outcomes

Secondary outcome variables will be analysed using the same modelling framework used for the primary outcome variable, although the study is not powered to analyse these outcomes nor moderation effects on the primary outcome. Dichotomous outcomes will be analysed using logistic regression. The results will be presented as estimates or odds ratios (with respective 95% confidence intervals [CI] and p-values), as appropriate. We will also compare the proportions of treatment responders and full or partial remitters in each group with logistic regression and odds ratio (95% CI). Between-group effect sizes will be estimated using Cohen's *d*.

8.2 SECONDARY OBJECTIVE 1: 6-MONTH DURABILITY OF THE TREATMENT EFFECTS

All trial participants will be naturalistically followed up and assessed up to 6 months after the end of the treatment. Linear mixed-effects models will be implemented for the primary and secondary outcome measures. As in the primary objective, for each outcome measure, the model will include fixed effects of time (3 time points: 1-month follow-up, 3-month follow-up, 6-month follow-up) and site, as well as a time by site interaction, and subject effects as a random intercept factor to account for the variances between and within participants. As above, site will be dropped from the model if its effects are not significant.

8.3 SECONDARY OBJECTIVE 2: HEALTH ECONOMIC EVALUATION

For the reporting of the health economic evaluation, we will follow the Consolidated Health Economic Evaluation Reporting Standards (CHEERS) guidelines. The health economic evaluation will compare ICBT and IRT at the primary end point (1-month follow-up).

The health economic evaluation will be performed from three different perspectives, with gradually increasing costs included for each perspective:

1) The first perspective is the health organisation payer perspective, which includes the direct costs of running the interventions for the clinic. This comprises personnel costs such as therapist time, administration time, IT platform maintenance, and other overhead costs.

2) The second perspective will additionally include other healthcare resource use outside the clinic for the participating children and adolescents, such as costs for medical appointments (outside the trial) and medications.
3) The third perspective will further comprise other societal costs, including productivity losses for the children/adolescents related to absenteeism and presenteeism from school and leisure activities, and productivity losses for the parents due to absenteeism from work.

The TiC-P (50) used in the current trial has been adapted for young people and caregivers/parents. It will be administered at each assessment point (except during the treatment) to collect information on frequencies of resource use for the children/adolescents and absenteeism from work for the parents. Resource use costs will be estimated by multiplying frequencies by national Swedish tariffs and market prices. Total costs for each group will be aggregated over the trial period.

For each of the three perspectives above, we will conduct two types of analyses:

1) A cost-effectiveness analysis, using responder rate as the outcome (59).
2) A cost-utility analysis using Quality Adjusted Life Years (QALYs) as the outcome (60).

Health gains in terms of QALYs will be estimated using CHU9D scores (49). CHU9D scores will be transformed into individual health-related quality of life scores, which will be used to estimate QALYs over the trial period, using the area under the curve method (61). We will compare costs and health outcomes between the two intervention arms over time using generalised linear models (62).

To ascertain whether the intervention is cost-effective, we will calculate an incremental cost-effectiveness ratio, as the ratio between the difference in costs and the difference in QALYs or responders between interventions, for each costing perspective and express them as cost per additional QALY and cost per responder. We will explore uncertainty around the cost and outcome estimates and perform relevant sensitivity analyses.

8.4 INTERIM ANALYSIS

There are no interim analyses planned.

9 ADVERSE EVENTS

9.1 ADVERSE EVENTS AND SERIOUS ADVERSE EVENTS

An Adverse Event (AE) is any untoward occurrence to a subject receiving an intervention which does not necessarily have a causal relationship with the intervention. An AE can be any unfavourable and unintended sign, symptom or disease temporally associated with receiving the intervention, whether or not is related to it.

Each AE is to be classified by the Principal Investigator as serious or non-serious. Seriousness is not defined by a medical term; it is a result or an outcome. An AE is defined as a Serious Adverse Event (SAE) if it:

- results in death
- is life-threatening
- requires inpatient hospitalization or prolongation of existing hospitalization
- results in persistent or significant disability or incapacity
- is otherwise considered medically significant by the investigator

9.2 EXPECTED ADVERSE EVENTS

Expected adverse events are based on previous research on ICBT for adults with BDD (30) on the previous face-to-face paediatric BDD trial ()(4), as well as on our previous pilot trial for children and adolescents with BDD (ClinicalTrials.gov: NCT05078320; (31). The expected adverse events in this trial are:

- Increased severity of BDD symptoms
- Increased distress or anxiety
- Lower mood
- Sleep disturbances
- Increased risk for self-harm, suicidal ideation, and/or suicide attempt
- Increased family conflict
- Alcohol/drug use
- Risky sexual behaviour (e.g., unprotected sex ending in pregnancy)

Adverse events will be closely monitored by the therapist and reported in accordance to the procedures outlined in this protocol (see Section 9.4).

9.3 RELATED ADVERSE EVENTS

The assessment of the relation between adverse events and the administration of the treatment is a decision based on all available information. The final decision is taken by the Principal Investigator. If the event is a result of the administration of any of the research procedures, then it will be classed as related.

All AE will be categorized in accordance with the definitions below:

- **Likely related:** Clinical event occurring within a reasonable time after administration of the intervention. It is unlikely that the event can be attributed to underlying disease or medications, but is most likely caused by the intervention.

- **Possibly related:** Clinical event occurring within a reasonable time after administration of the intervention. The event could be explained by the intervention, but there is insufficient information to determine the relationship. The event could be explained by an underlying disease or medications.
- **Not related:** Clinical event that is not reasonably related to the intervention. The event is unlikely related to the intervention and can be explained by medications or underlying disease.

9.4 HANDLING OF ADVERSE EVENTS

All adverse events will be noted by the trial coordinator in a specific log (including date, recorded clinical symptoms, and a brief description of the event), which in turn will be shared with the Karolinska Trial Alliance. The research team could be notified about adverse events in numerous ways, including through direct (text or telephone) communication with the participant, and completion of the adverse events questionnaire. Regular meetings ("ward rounds") will be held within the research team where treatment progress and potential adverse events are discussed. High scores on SMFQ (depressive symptoms), with the additional suicide item, and the DSHI-Y-7 (self-harm) might also be indicators of adverse events. Scores ≥13 points on the SMFQ, or scores of 2 ("I have had thoughts about death and of taking my life, but would never do it") or 3 ("I have had thoughts about death and of taking my life") points on the suicide item, will automatically raise a flag in the BASS system that will directly notify members of the research team to follow this up (via the telephone) with the participant or her/his caregivers. It will be recorded as an AE if it is greater than their baseline score.

The listed sources will be combined and AEs will be summarized by number (frequencies and percentages), nature, severity, and whether they are expected or unexpected. If the event has been listed under 9.2 in the study protocol, the event will be classed as expected. If the event is not listed, then it will be classed as unexpected. If there are any SAEs, the trial coordinator and the Principal Investigator will judge whether these are related to the treatments provided. Events will be considered as potentially treatment-related up to the 6-month follow-up.

Appropriate action will be taken in the case of SAE, making sure the participant will get in contact with suitable health care services. In the event of a SAE, the Principal Investigator will immediately notify the Sponsor and the monitor (Karolinska Trial Alliance), then they will work together to identify the extent of the SAE and determine what urgent safety measures are required.

10 DATA SHARING

We will potentially share trial data with other researchers around the world. The use of the data will aim to advance the field of BDD, for instance, combining treatment outcome data for individual participant data meta-analyses. Shared data will be pseudonymised. The possibility of future data sharing is mentioned in the informed consent form.

11 ETHICAL CONSIDERATIONS

Both treatments (ICBT and IRT) differ from their respective face-to-face versions only in the format and mode of delivery, but still contain the same evidence-based components. While there is some preliminary evidence to suggest that ICBT may be efficacious (26,27,30,31), at present, there is no evidence that it will be superior to IRT in reducing BDD symptom severity. Thus, the trial fulfils the principle of equipoise. Nonetheless, our hypothesis is that ICBT may indeed be superior to IRT. For that reason, all individuals randomized to the comparator (IRT) will be offered cross-over after the controlled period ends at the 1-month follow-up.

Verbal information about the study and written informed consent forms will be provided to the child/adolescent and all legal guardians prior to baseline assessment. All participant families will be volunteers, competent to provide informed consent. Participants may withdraw from the trial at any time.

BDD has previously been associated with suicidality and patient safety will be a high priority in this study. Participants will complete measures of depression and suicide weekly allowing for these symptoms to be monitored throughout the intervention. A thorough diagnostic and mental health status examination before enrolment of participants will prevent patients with more immediate needs or risks from being included in the study. Children and adolescents who have attempted suicide in the year prior to the assessment will be excluded to reduce the risk of suicide attempts and increase patient safety during the trial. These patients will be referred to other services or offered alternative treatment at the clinic, as appropriate.

This study will be conducted within regular health care and all participants will be enrolled as patients at one of the three clinics. As patients, they will be followed according to the ordinary clinical routines. We will also have weekly meetings discussing patients, including a child and adolescent psychiatrist. Specific procedures will be in place to handle potential acute situations (e.g., self-harm) and clinical personnel will be able to quickly refer children/adolescents in need of more help to their local mental health care services or emergency services if needed.

It is unlikely that participants will experience any serious adverse events during this trial as a result of receiving either of the two treatments. Adverse events will be carefully monitored throughout the trial. There will be close liaison between the participant and the therapist throughout the trial so that any adverse reactions can be noted. All participants that experience a serious adverse event will be followed-up until the event is resolved. Some participants are expected to experience adverse events that are related to their participation in the trial, such as increased stress or low mood. However, these reactions are common when young people undergo psychological treatment and usually transient.

Participants may worry about computer safety and confidentiality. To prevent this, families will receive information about the risks and precautions that are being taken when using communication technology (e.g., encrypted server technology and double authentication with password and SMS-code). Swedish and international rules and regulations will be followed. Our research team has extensive experience and knowledge of the approval processes, systems, and good practice guidelines for clinical research in Sweden and internationally.

Each participant will be assigned a unique trial identification number at the point of inclusion. This number will be recorded on all paper datasheets and in the electronic trial database. All data will be kept secure at all times and maintained in accordance with the requirements of Good Clinical Practice regulations. The Karolinska Institutet Electronic Lab Notebook (ELN) will be used to log all significant decisions during the trial.

Overall, the risks for the participants are considered to be limited and temporary. The potential benefits for the participants and the scientific value of the study far outweigh the potential risks of participation.

12 IMPLICATIONS

The current evidence base for the treatment of BDD in young people is very limited. This study will contribute to the evidence base by evaluating the relative efficacy, durability, and cost-effectiveness of two psychological treatments for the disorder, thus advancing the field. This trial could add a potentially cost-effective way to deliver evidence-based psychological treatment for BDD and could increase the availability to such treatments by breaking down geographical and other barriers to access them.

13 FUNDING

The trial has received funding from Region Stockholm (ALF Medicin), Region Skåne (ALF Projekt), Folksams forskningsstiftelse, the International OCD Foundation, the Jane och Dan Olssons Stiftelse för Vetenskapliga ändamål, Centre for Psychiatry Research at Region Stockholm/Karolinska Institutet, Fredrik och Ingrid Thurings stiftelse, Crafoordska stiftelsen, Stiftelsen Söderström - Königska sjukhemmet, Stiftelsen Lindhaga, and Drottning Silvias Jubileumsfond. Additional funding is being sought.

14 PUBLICATION PLANS

We plan to publish one or two primary papers. The main paper will report on the efficacy of the interventions (up to the 1-month follow-up [primary endpoint]) and follow-up data (from the 1-month follow-up to the 6-month follow-up). The cost-effectiveness results may be included in the main report or published separately. Additional papers may include analysis of predictors and moderators of treatment outcome. All manuscripts will be submitted to general or specialist medical and psychological science journals.

REFERENCES

1. Veale D, Gledhill LJ, Christodoulou P, Hodsoll J. Body dysmorphic disorder in different settings: A systematic review and estimated weighted prevalence. Body Image. 2016 Sep 1;18:168–86.

2. Kocsis RN. Diagnostic and Statistical Manual of Mental Disorders: Fifth Edition (DSM-5). Int J Offender Ther Comp Criminol. 2013 Dec 1;57(12):1546–8.

3. Phillips KA, Didie ER, Menard W, Pagano ME, Fay C, Weisberg RB. Clinical features of body dysmorphic disorder in adolescents and adults. Psychiatry Res. 2006 Jan 1;141(3):305–14.

4. Mataix-Cols D, Fernández de la Cruz L, Isomura K, Anson M, Turner C, Monzani B, et al. A Pilot Randomized Controlled Trial of Cognitive-Behavioral Therapy for Adolescents With Body Dysmorphic Disorder. J Am Acad Child Adolesc Psychiatry. 2015 Jan 1;54(11):895–904.

5. Enander J, Ivanov V, Mataix-Cols D, Kuja-Halkola R, Ljotsson B, Lundstrom S, et al. Prevalence and heritability of body dysmorphic symptoms in adolescents and young adults: a population-based nationwide twin study. Psychol Med. 2018 Jan 1;48(16):2740–7.

6. Schneider SC, Turner CM, Mond J, Hudson JL. Prevalence and correlates of body dysmorphic disorder in a community sample of adolescents. Aust N Z J Psychiatry. 2017 Jun;51(6):595–603.

7. Bjornsson AS, Didie ER, Grant JE, Menard W, Stalker E, Phillips KA. Age at onset and clinical correlates in body dysmorphic disorder. Compr Psychiatry. 2013 Oct 1;54(7):893–903.

8. Rautio D, Jassi A, Krebs G, Andren P, Monzani B, Gumpert M, et al. Clinical characteristics of 172 children and adolescents with body dysmorphic disorder. Eur Child Adolesc Psychiatry. 2022 Jan 1;31(1):133–44.

9. Krebs G, Fernández de la Cruz L, Monzani B, Bowyer L, Anson M, Cadman J, et al. Long-Term Outcomes of Cognitive-Behavioral Therapy for Adolescent Body Dysmorphic Disorder. Behav Ther. 2017 Jan 1;48(4):462–73.

10. Albertini RS, Phillips KA. Thirty-Three Cases of Body Dysmorphic Disorder in Children and Adolescents. J Am Acad Child Adolesc Psychiatry. 1999 Jan 1;38(4):453–9.

11. Obsessive-compulsive disorder: Evidence Update September 2013: A summary of selected new evidence relevant to NICE clinical guideline 31 ‘Obsessive-compulsive disorder: core interventions in the treatment of obsessive-compulsive disorder and body dysmorphic disorder’ (2005). 2013;

12. Veale D, Phillips KA, Neziroglu F. Challenges in Assessing and Treating Patients with Body Dysmorphic Disorder and Recommended Approaches. In: Phillips KA, Phillips KA, editors. Body Dysmorphic Disorder: Advances in Research and Clinical Practice [Internet]. Oxford University Press; 2017 [cited 2023 Mar 26]. p. 0. Available from: https://doi.org/10.1093/med/9780190254131.003.0024

13. Riktlinjer till stöd för bedömning och behandling. Vol. 2015. Stockholm, Sweden: Barn och ungdomspsykiatrin Sll.;

14. Harrison A, Fernández de La Cruz L, Enander J, Radua J, Mataix-Cols D. Cognitive-behavioral therapy for body dysmorphic disorder: A systematic review and meta-analysis of randomized controlled trials. Clin Psychol Rev. 2016 Jan 1;48:43–51.

15. Wilhelm S, Phillips KA, Greenberg JL, O'Keefe SM, Hoeppner SS, Keshaviah A, et al. Efficacy and posttreatment effects of therapist-delivered cognitive behavioral therapy vs supportive psychotherapy for adults with body dysmorphic disorder: A randomized clinical trial. JAMA Psychiatry. 2019 Apr;76(4):363–73.

16. Rautio G Daniel Martina, Jassi A, Krebs G, Flygare A Oskar Per, Monzani P Benedetta Lauren, Jansson-Fröjmark L Markus Tobias, et al. Effectiveness of Multimodal Treatment for Young People With Body Dysmorphic Disorder in Two Specialist Clinics. Behav Ther. 2022 Sep 1;53(5):1037–49.

17. Krebs G, Turner C, Heyman I, Mataix-Cols D. Cognitive behaviour therapy for adolescents with body dysmorphic disorder: A case series. Behav Cogn Psychother. 2012 Jul;40(4):452–61.

18. Greenberg JL, Mothi SS, Wilhelm S. Cognitive-Behavioral Therapy for Adolescent Body Dysmorphic Disorder: A Pilot Study. Behav Ther. 2016 Mar 1;47(2):213–24.

19. Wilver NL, Cougle JR. An Internet-based controlled trial of interpretation bias modification versus progressive muscle relaxation for body dysmorphic disorder. J Consult Clin Psychol. 2019 Mar;87(3):257–69.

20. Andersson G, Titov N, Dear B, Rozental A, Carlbring P. Internet-delivered psychological treatments: from innovation to implementation. World Psychiatry Off J World Psychiatr Assoc WPA. 2019 Jan 1;18(1):20–8.

21. Aspvall K, Andersson E, Melin K, Norlin L, Eriksson V, Vigerland S, et al. Effect of an Internet-Delivered Stepped-Care Program vs In-Person Cognitive Behavioral Therapy on Obsessive-Compulsive Disorder Symptoms in Children and Adolescents: A Randomized Clinical Trial. JAMA. 2021 Jan 1;325(18):1863–73.

22. Jolstedt M, Vigerland S, Mataix-Cols D, Ljotsson B, Wahlund T, Nord M, et al. Long-term outcomes of internet-delivered cognitive behaviour therapy for paediatric anxiety disorders: towards a stepped care model of health care delivery. Eur Child Adolesc Psychiatry. 2021 Jan 1;30(11):1723–32.

23. Hedman E, Ljotsson B, Lindefors N. Cognitive behavior therapy via the Internet: a systematic review of applications, clinical efficacy and cost-effectiveness. Expert Rev Pharmacoecon Outcomes Res. 2012 Jan 1;12(6):745–64.

24. Andersson G, Cuijpers P, Carlbring P, Riper H, Hedman E. Guided Internet-based vs. face-to-face cognitive behavior therapy for psychiatric and somatic disorders: a systematic review and meta-analysis. World Psychiatry Off J World Psychiatr Assoc WPA. 2014 Jan 1;13(3):288–95.

25. Vigerland S, Lenhard F, Bonnert M, Lalouni M, Hedman E, Ahlen J, et al. Internet-delivered cognitive behavior therapy for children and adolescents: A systematic review and meta-analysis. Clin Psychol Rev. 2016 Jan 1;50:1–10.

26. Enander J, Ivanov VZ, Andersson E, Mataix-Cols D, Ljótsson B, Rück C. Therapist-guided, Internet-based cognitive-behavioural therapy for body dysmorphic disorder (BDD-NET): a feasibility study. BMJ Open. 2014 Sep 25;4(9):e005923.

27. Gentile A, La Lima C, Flygare O, Enander J, Wilhelm S, Mataix-Cols D, et al. Internet-based, therapist-guided, cognitive-behavioural therapy for body dysmorphic disorder with global eligibility for inclusion: an uncontrolled pilot study. BMJ Open. 2019 Jan 1;9(3):e024693.

28. Enander J, Ljotsson B, Anderhell L, Runeborg M, Flygare O, Cottman O, et al. Long-term outcome of therapist-guided internet-based cognitive behavioural therapy for body dysmorphic disorder (BDD-NET): a naturalistic 2-year follow-up after a randomised controlled trial. BMJ Open. 2019 Jan 1;9(1):e024307.

29. Wilhelm S, Weingarden H, Greenberg JL, McCoy TH, Ladis I, Summers BJ, et al. Development and Pilot Testing of a Cognitive-Behavioral Therapy Digital Service for Body Dysmorphic Disorder. Behav Ther. 2020 Jan;51(1):15–26.

30. Wilhelm S, Weingarden H, Greenberg JL, Hoeppner SS, Snorrason I, Bernstein EE, et al. Efficacy of App-Based Cognitive Behavioral Therapy for Body Dysmorphic Disorder with Coach Support: Initial Randomized Controlled Clinical Trial. Psychother Psychosom. 2022 Jul;91(4):277–85.

31. Rautio D. et al. (in preparation). Internet-delivered cognitive-behavior therapy for adolescents with body dysmorphic disorder: an open feasibility trial.

32. Lenhard F, Andersson E, Mataix-Cols D, Ruck C, Vigerland S, Hogstrom J, et al. Therapist-Guided, Internet-Delivered Cognitive-Behavioral Therapy for Adolescents With Obsessive-Compulsive Disorder: A Randomized Controlled Trial. J Am Acad Child Adolesc Psychiatry. 2017 Jan 1;56(1):10–9.

33. Nordh M, Wahlund T, Jolstedt M, Sahlin H, Bjureberg J, Ahlen J, et al. Therapist-Guided Internet-Delivered Cognitive Behavioral Therapy vs Internet-Delivered Supportive Therapy for Children and Adolescents With Social Anxiety Disorder: A Randomized Clinical Trial. JAMA Psychiatry. 2021 Jan 1;78(7):705–13.

34. Tolin, D. F., Sain, K. S., Davis, E., Gilliam, C., Hannan, S. E., Springer, K. S., Stubbing, J., George, J. R., Jean, A., Goldblum, R., Katz, B. W., Everhardt, K., Darrow, S., Ohr, E. E., Young, M. E., & Serchuk, M. D. (2023). The DIAMOND-KID: Psychometric properties of a structured diagnostic interview for DSM-5 anxiety, mood, and obsessive-compulsive and related disorders in children and adolescents. *Assessment*, 10731911221143994

35. Structured Clinical Interview for DSM Disorders (SCID) [Internet]. Columbia University Department of Psychiatry. 2018 [cited 2023 Mar 26]. Available from: https://www.columbiapsychiatry.org/research/research-areas/services-policy-and-law/structured-clinical-interview-dsm-disorders-scid

36. Phillips KA, Hollander E, Rasmussen SA, Aronowitz BR. A severity rating scale for body dysmorphic disorder: Development, reliability, and validity of a modified version of the Yale-Brown Obsessive Compulsive Scale. Psychopharmacol Bull. 1997;33(1):17–22.

37. Monzani B, Fallah D, Rautio D, Gumpert M, Jassi A, de la Cruz LF, et al. Psychometric evaluation of the yale-brown obsessive-compulsive scale modified for body dysmorphic disorder for adolescents (bdd-ybocs-a). Child Psychiatry Hum Dev. 2022 Jun 9;

38. Fernández de la Cruz L, Enander J, Rück C, Wilhelm S, Phillips K, Steketee G, et al. Empirically defining treatment response and remission in body dysmorphic disorder. Psychol Med. 2021 Jan 1;51(1):83–9.

39. Busner J, Targum SD. The Clinical Global Impressions Scale: Applying a research tool in clinical practice. Psychiatry. 2007 Jul;4(7):28–35.

40. Shaffer D, Gould MS, Brasic J, Ambrosini P, Fisher P, Bird H, et al. A children's global assessment scale (CGAS). Arch Gen Psychiatry. 1983 Nov;40(11):1228–31.

41. Veale D, Eshkevari E, Kanakam N, Ellison N, Costa A, Werner T. The Appearance Anxiety Inventory: Validation of a process measure in the treatment of body dysmorphic disorder. Behav Cogn Psychother. 2014 Sep;42(5):605–16.

42. Rhew IC, Simpson K, Tracy M, Lymp J, McCauley E, Tsuang D, et al. Criterion validity of the Short Mood and Feelings Questionnaire and one- and two-item depression screens in young adolescents. Child Adolesc Psychiatry Ment Health. 2010 Feb 9;4.

43. Spitzer RL, Kroenke K, Williams JBW, Löwe B. A brief measure for assessing generalized anxiety disorder: The GAD-7. Arch Intern Med. 2006 May 22;166(10):1092–7.

44. Gratz K. Measurement of deliberate self-harm: Preliminary data on the deliberate self-harm inventory. J Psychopathol Behav Assess. 2001 Dec 1;23(4):253–63.

45. Knight JR, Shrier LA, Bravender TD, Farrell M, Vander Bilt J, Shaffer HJ. A new brief screen for adolescent substance abuse. Arch Pediatr Adolesc Med. 1999 Jun;153:591–6.

46. Källmén H, Berman AH, Jayaram-Lindström N, Hammarberg A, Elgán TH. Psychometric Properties of the AUDIT, AUDIT-C, CRAFFT and ASSIST-Y among Swedish Adolescents. Eur Addict Res. 2019 Jan 1;25(2):68–77.

47. Jassi A, Lenhard F, Krebs G, Gumpert M, Jolstedt M, Andren P, et al. The Work and Social Adjustment Scale, Youth and Parent Versions: Psychometric Evaluation of a Brief Measure of Functional Impairment in Young People. Child Psychiatry Hum Dev. 2020 Jan 1;51(3):453–60.

48. Mundt JC, Marks IM, Shear MK, Greist JM. The Work and Social Adjustment Scale: A simple measure of impairment in functioning. Br J Psychiatry. 2002 May;180(5):461–4.

49. Lindvall K, Vaezghasemi M, Feldman I Docent, 1951, Ivarsson A, Stevens KJ, Petersen S. Feasibility, reliability and validity of the health-related quality of life instrument Child Health Utility 9D (CHU9D) among school-aged children and adolescents in Sweden. Health Qual Life Outcomes. 2021 Jan 1;19(1).

50. Bouwmans C, Jong KD, Timman R, Zijlstra-Vlasveld M, Van der Feltz-Cornelis C, Tan SS, et al. Feasibility, reliability and validity of a questionnaire on healthcare consumption and productivity loss in patients with a psychiatric disorder (TiC-P). BMC Health Serv Res. 2013 Jun 15;13:UNSP 217.

51. Andrusyna TP, Tang TZ, DeRubeis RJ, Luborsky L. The factor structure of the Working Alliance Inventory in cognitive-behavioral therapy. J Psychother Pract Res. 2001 Sum;10(3):173–8.

52. Larsen DL, Attkisson CC, Hargreaves WA, Nguyen TD. Assessment of client/patient satisfaction: Development of a general scale. Eval Program Plann. 1979;2:197–207.

53. Simpson HB, Maher M, Page JR, Gibbons CJ, Franklin ME, Foa EB. Development of a Patient Adherence Scale for Exposure and Response Prevention Therapy. Behav Ther. 2010 Jan 1;41(1):30–7.

54. Lenhard F, Mitsell K, Jolstedt M, Vigerland S, Wahlund T, Nord M, et al. The Internet Intervention Patient Adherence Scale for Guided Internet-Delivered Behavioral Interventions: Development and Psychometric Evaluation. J Med Internet Res. 2019 Oct;21(10):N.PAG-N.PAG.

55. Randomize.net - Clinical Trial Randomization Service [Internet]. [cited 2023 Feb 18]. Available from: https://randomize.net/

56. Karolinska Trial Alliance - KTA [Internet]. 2023 [cited 2023 Feb 18]. Available from: https://www.karolinska.se/kta

57. Freeman J, Sapyta J, Garcia A, Compton S, Khanna M, Flessner C, et al. Family-based treatment of early childhood obsessive-compulsive disorder: The Pediatric Obsessive-Compulsive Disorder Treatment Study for Young Children (POTS Jr)—A randomized clinical trial. JAMA Psychiatry. 2014 Jun;71(6):689–98.

58. Moher D, Hopewell S, Schulz KF, Montori V, Gøtzsche PC, Devereaux P j., et al. CONSORT 2010 explanation and elaboration: Updated guidelines for reporting parallel group randomised trials. Int J Surg. 2012 Jan 1;10(1):28–55.

59. Mataix-Cols D, Fernández de la Cruz L, Nordsletten A, Lenhard F, Isomura K, Simpson H. Towards an international expert consensus for defining treatment response, remission, recovery and relapse in obsessive-compulsive disorder. World Psychiatry Off J World Psychiatr Assoc WPA. 2016 Jan 1;15(1):80.

60. Michael F. Drummond, Mark J. Sculpher, Karl Claxton, Greg L. Stoddart, George W. Torrance. Methods for the Economic Evaluation of Health Care Programmes. Oxford: OUP Oxford; 2015. (Oxford Medical Publications; vol. Fourth edition. Michael F. Drummond, Mark J. Sculpher, Karl Claxton, Greg 1. Stoddart, George W. Tor).

61. Matthews JNS, Altman DG, Campbell MJ, Royston P. Analysis Of Serial Measurements In Medical Research. BMJ. 1990 Jan 27;300(6719):230–5.

62. Barber J, Thompson S. Multiple regression of cost data: use of generalised linear models. J Health Serv Res Policy. 2004 Oct 1;9(4):197–204.
