# Supplementary material for: Efficacy and cost-effectiveness of Internet-delivered cognitive-behavior therapy for children and adolescents with body dysmorphic disorder: study protocol for a randomized controlled trial
Source: Trials. 2026 Aug 31;27:557. doi: 10.1186/s13063-026-09974-z (PMC13527960; doi:10.1186/s13063-026-09974-z)
Supplement: Supplementary file 2 [file 13063_2026_9974_MOESM4_ESM.pdf]

BESLUT

2023-04-26

Sökande forskningshuvudman
Karolinska Institutet

Forskare som genomför projektet
Lorena Fernández de la Cruz

Projekttitel
En randomiserad kontrollerad studie om internetförmedlad kognitiv beteendeterapi för ungdomar med dysmorfofobi.

Uppgifter om ansökan
Ansökan inkom till Etikprövningsmyndigheten 2023-03-31 och blev valid 2023-04-06.

Etikprövningsmyndigheten beslutar enligt nedan.

BESLUT

Etikprövningsmyndigheten godkänner den forskning som anges i ansökan.

Etikprövningsmyndigheten
2023-02193-01-404603
2023-05-08

På Etikprövningsmyndighetens vägnar

Anna Lander
Ordförande

Beslutet har fattats av följande personer:

Ordförande
Anna Lander (Rådman)

Ledamöter med vetenskaplig kompetens
Cecilia Gunnarsson (Hereditära Kardiovaskulära sjukdomar, genetik, syndrom, vetenskaplig sekreterare)
Martin Hägglund (fysioterapi, epidemiologi, vetenskaplig sekreterare)
Karin Åkesson (Pediatrik, föredragande)
Erik Lexne (Psykiatri)
Preben Kirkegaard Kjölhede (Obstetrik/gynekologi. Gynekologisk onkologi.)
Ulrika Müssener (Folkhälsa)
Camilla Nilsberth (Geriatrik, Neurobiologi)
Håkan Olausson (Klinisk neurofysiologi)
Per Sandström (Kirurgi)

Etikprövningsmyndigheten
2023-02193-01-404603
2023-05-08

Ledamöter som företräder allmänna intressen
Martina Aronsson
Per-Arne Fall
Fredrik Hasselgren
Bertil Nilsson
Stefan Redéen

Beslutet sänds till
Ansvarig forskare: Lorena Fernández de la Cruz
Forskningshuvudmannens företrädare: Mats Olsson
