# Supplementary material for: Efficacy and cost-effectiveness of Internet-delivered cognitive-behavior therapy for children and adolescents with body dysmorphic disorder: study protocol for a randomized controlled trial
Source: Trials. 2026 Aug 31;27:557. doi: 10.1186/s13063-026-09974-z (PMC13527960; doi:10.1186/s13063-026-09974-z)
Supplement: Supplementary file 4 [file 13063_2026_9974_MOESM3_ESM.docx]

**Statistical and health economics analysis plan**

**Version 1.0, 2026-01-13**

1. **Introduction**

**1.1 Purpose**

This plan contains details of the main statistical analyses for the “efficacy and cost-effectiveness of Internet-delivered cognitive-behavior therapy (ICBT) for children and adolescents with body dysmorphic disorder (BDD)” randomized controlled trial (RCT). These analyses are pre-specified in order for them not to be influenced by the collected trial data after unblinding. The plan describes the statistical analysis of the longitudinal clinical outcomes (efficacy) and the health economic evaluation.

The plan does not preclude the undertaking of further post-hoc or exploratory analyses, although the results of such analyses should be interpreted with caution. Furthermore, the plan does not preclude the modification of any part of the trial analysis, should particular situations arise in which such adaptation is deemed necessary. Such adaptations, if performed, will be documented in the Karolinska Institutet Electronic Lab Notebook (ELN) and clearly labelled as post-hoc in the published paper.

1.2 **Trial registration and protocol**

The trial was prospectively registered at ClinicalTrials.gov (NCT06262412). Full information on the trial design, interventions, assessment points, measures, etc. is described in the full study protocol (current version 1.4). This SAP should be interpreted in the context of the information provided in the full study protocol.

2. **Study aims**
   2.1 **Primary aim**

Aim #1: To determine the clinical efficacy of therapist-guided ICBT for BDD in reducing BDD symptom severity (as measured by the Yale-Brown Obsessive Compulsive Scale modified for BDD, Adolescent version [BDD-YBOCS-A]) [1, 2] in children and adolescents with BDD, compared with a comparator consisting of therapist-guided, Internet-delivered relaxation training (IRT) for BDD. The primary endpoint is the 1-month follow-up post-treatment. We hypothesize that ICBT will be superior to IRT in reducing BDD symptom severity at the primary endpoint in children and adolescents with BDD.

2.2 **Secondary aims**

Aim #2: To establish the 6-month durability of the treatment effects in a naturalistic follow-up. We hypothesize that participants receiving ICBT will maintain their treatment gains at the 6-month follow-up.

Aim #3: To conduct a health-economic evaluation of ICBT for BDD at the primary endpoint from a health organization payer, healthcare resource use, and societal perspective. We hypothesize that ICBT for BDD will be more cost-effective than IRT.

3. **Reporting**

We will follow the Consolidated Standards of Reporting Trials (CONSORT) 2025 guidelines [3] when reporting clinical outcomes, and the Consolidated Health Economic Evaluation Reporting Standards (CHEERS) 2022 guidelines [4] when reporting health economic results.

We plan to publish one or two primary papers. The main paper will report on the efficacy of the interventions (up to the 1-month follow-up [primary endpoint]) and follow-up data (from the 1-month follow-up to the 6-month follow-up). The cost-effectiveness results may be included in the main report or published separately. Additional papers may include analysis of predictors and moderators of treatment outcome (research questions and aims yet to be determined). All these analyses will be considered post-hoc. All manuscripts will be submitted to general or specialist medical and psychological science journals.

4. **Statistical analysis of clinical outcomes**
   4.1 **Responsibility**

Statistical analyses relating to the clinical efficacy and 6-month durability aims will be conducted in-house by the trial coordinator at the Stockholm site (Anita Birovecz), blinded to group allocation, and then independently replicated by personnel at the Biostatistics Core Facility at Karolinska Institutet ([www.biostatcore.ki.se](http://www.biostatcore.ki.se)), blinded to both aims/hypothesis and group allocation. The economic evaluation will be conducted under the supervision of co-investigator Erik Andersson at Karolinska Institutet, blinded to group allocation.

4.2 **Organization of data and analyses**

Assessments are conducted at baseline, mid-treatment, post-treatment, and 1-, 3- and 6-month post-treatment, depending on the measure. Analyses will be performed in two stages. Stage 1 will contain data collected between baseline and the 1-month follow-up (the primary endpoint). Stage 2 will contain data collected at all assessment points, from baseline to the 6-month follow-up. The two-stage analysis will require two corresponding database locks. The primary outcome analysis will be carried out in stage 1 (corresponding to aim #1).

The primary data analysis will be conducted after the last participant has completed the 1-month follow-up and all collected data have been entered into the trial database. Assessors will however remain blind to the individual participants’ treatment allocations until data for the final 6-month follow-up assessment has been collected. The statistical analysis plan will be finalized, approved, and published prior to unblinding.

Prior to each database lock, basic checks will be performed on the collected data to ensure accuracy. Anita Birovecz, who will be blind to group allocation, will conduct these checks. Each baseline demographic variable and outcome variables (both primary and secondary) will be checked for:

- Missing values
- Values outside an acceptable range
- Other inconsistencies

If missing values or other inconsistencies are found, Birovecz will investigate them and compare the data in the databases with the source data (if available). The data will then be either corrected or deemed as missing, as appropriate. The database will then be locked for data changes by Birovecz. To ensure blinding, a researcher not involved in the analysis will create provisional dummy variables in place for the group allocation and participant ID variables, so that the researchers performing the analysis are not influenced by the knowledge of group allocation.

The statistical programming code to be used for the primary analysis (aim #1) will be prepared prior to unblinding. The primary analysis (aim #1) will be performed by Birovecz and independently replicated by personnel at the Biostatistics Core Facility at Karolinska Institutet, led by co-investigator Bottai. Birovecz will be blinded to participant allocation, while the personnel at the Biostatistics Core Facility will be blinded to both aims/hypotheses and participant allocation.

4.3 **Interim analyses**

No interim analyses are planned.

4.4 **Recruitment and retention**

A CONSORT 2025 flow diagram [3] will be presented to provide a detailed description of the participant flow throughout the study, including number of participants screened, reasons for exclusion, number of participants randomized to each group, and number of participants assessed at each follow-up point. See CONSORT flow diagram in the protocol for the exact content that will be presented.

4.5 **Description of demographic variables at baseline**

The demographic information collected at baseline will be summarized and presented in a table, by randomization arm. Categorical variables will be reported as counts and percentages. Continuous variables will be summarized as means, medians, and interquartile ranges, as appropriate. According to CONSORT recommendations, no statistical tests will be performed to assess baseline differences between study arms [3].

4.6 **Protocol deviations**

Protocol deviations that could impact on the results of the analyses (e.g., concomitant psychological treatment or medication) will be described in detail. Further analyses taking such deviations into account (e.g., sensitivity analyses excluding affected data points) may be performed, depending on the amount and extent of the deviations. These analyses will be considered post-hoc.

4.7 **Primary outcome analysis**

The primary outcome is BDD symptom severity, as measured by the BDD-YBOCS, at the primary endpoint (1-month follow-up). The primary outcome analysis will be based on all available data up to this time point and conducted according to the intention-to-treat (ITT) principle.

All the randomized participants will be included in a linear mixed-effect model to estimate the difference in between-group means over time. Linear mixed models use all available data, can properly account for correlation between repeated measurements on the same subject, have greater flexibility to model time effects, and can handle missing data. The model will include fixed effects of time (baseline, post-treatment, and 1-month follow-up), treatment group (ICBT or IRT), site (Region Stockholm, Västra Götalandsregionen, or Region Skåne), and interaction effects of group by time, group by site, site by time, and group by site by time, as well as random intercept to account for individual differences. If the effects of site are not statistically significant, this variable will be dropped from the model to obtain a more parsimonious model. The primary source of inference will be the between-group difference at the primary end point (1-month follow up), estimated from the group by time interaction. Estimated treatment effects will be reported with the accompanying 95% confidence intervals (CI) and *p*-value. The alpha level of 0.05 will be used as the threshold for statistical significance throughout all trial analyses.

*4.7.1 Model checking*

The statistical model for the primary outcome analysis includes an assumption that the subject-specific random intercepts are normally distributed. This assumption will be checked through the construction of appropriate histograms and normal quantile plots. If these plots suggest that residuals are not normally distributed, then appropriate transformations of the primary outcome or application of the bootstrap method will be considered.

*4.7.2 Missing data*

Potential selection bias caused by missing data will be investigated by comparing the baseline characteristics of participants with and without missing values on the BDD-YBOCS-A using logistic regression. Depending on the number of missing values, predictors of missingness will be identified. We will then perform a sensitivity analysis by including any predictors of missingness as explanatory variables in the primary outcome model. Analyses investigating the impact of missing data will be considered supportive.

4.8 **Analysis of secondary outcomes**

Secondary outcome variables (see study protocol for a full list) will be analyzed using the same modelling framework used for the primary outcome variable, although the study is not powered to analyze these outcomes or moderation effects on the primary outcome. Dichotomous outcomes will be analyzed using logistic regression. The results will be presented as estimates or odds ratios (with respective 95% confidence intervals [CIs] and p-values), as appropriate. We will also compare the proportions of treatment responders and full or partial remitters in each group with logistic regression and odds ratio (95% CI).

4.9 **Long-term follow-up analysis**

All trial participants will be naturalistically followed up (i.e., if needed, participants will be able to seek other kinds of help after the primary endpoint at the 1-month follow-up) and assessed up to 6 months after the end of the treatment. The blind assessors will remain blind for the individual participants’ treatment allocations until data for the final 6-month follow-up assessment have been collected. These naturalistic long-term follow-up analyses will be carried out in stage 2. The analyses will be performed on the second locked database.

Linear mixed-effects models will be implemented to evaluate in a within-group analysis whether the potential treatment effects demonstrated for ICBT at the 1-month follow-up in stage 1 are sustained at the 6-month follow-up. As in the primary objective, for each outcome measure, the model will include fixed effects of time (three time points: 1-month follow-up, 3-month follow-up, 6-month follow-up) and site, as well as a time by site interaction, and a random intercept to account for individual differences.

Additionally, we will also enter all available assessment points to a separate regression model to investigate whether there are significant between-group effects at the 6-month follow-up. Results from such between-group analyses, however, will be interpreted with caution, given the risk of bias associated with long follow-up periods (e.g., patients with poor short-term outcomes are more prone to receiving other potentially effective treatments during the follow-up period).

4.10 **Subgroup analysis**

No subgroup analyses are planned.

4.11 **Effect sizes**

For all previous analyses, for mean comparisons, the magnitude of the treatment effects will additionally be presented as standardized between-group effect sizes using Cohen’s *d*. This will be used to enable comparisons with previous trials in the field.

4.12 **Adverse events**

The adverse events will be summarized by number (frequencies and percentages), nature, severity, and whether they are expected or unexpected. If there are any serious adverse events, the Principal Investigator will judge whether these are related to the treatments provided. No statistical tests comparing the treatment groups will be performed. See the full study protocol for detailed information regarding the data collection of adverse events, including definitions to classify them as expected or unexpected.

5. **Health economic evaluation**
   5.1 **Responsibility**

The economic evaluation will be conducted under the supervision of co-investigator Andersson at Karolinska Institutet, who will be blinded to group allocation.

5.2 **Analysis**

For the reporting of the health economic evaluation, we will follow the Consolidated Health Economic Evaluation Reporting Standards (CHEERS) 2022 guidelines [4]. The health economic evaluation will compare ICBT and IRT at the primary end point (1-month follow-up) from three different perspectives, with gradually increasing costs included for each perspective:

1) The first perspective is the health organization payer perspective, which includes the direct costs of running the interventions for the clinic. This comprises personnel costs such as therapist time, administration time, IT platform maintenance, and other overhead costs.
2) The second perspective will additionally include other healthcare resource use outside the clinic for the participating children and adolescents, such as costs for medical appointments (outside the trial) and medications.
3) The third perspective will further comprise other societal costs, including productivity losses for the children/adolescents related to absenteeism and presenteeism from school and leisure activities, and productivity losses for the parents due to absenteeism from work.

The Treatment Inventory of Costs in Patients with Psychiatric Disorders (TIC-P) [5] used in the current trial has been adapted for young people and caregivers/parents. It will be administered at each assessment point (except during the treatment) to collect information on frequencies of resource use for the children/adolescents and absenteeism from work for the parents. Resource use costs will be estimated by multiplying frequencies by national Swedish tariffs and market prices. Total costs for each group will be aggregated over the trial period.

For each of the three perspectives above, we will conduct two types of analyses:

1) A cost-effectiveness analysis, using responder rate as the outcome.

2) A cost-utility analysis using Quality Adjusted Life Years (QALYs) as the outcome.

Health gains in terms of QALYs will be estimated using Child Health Utility 9D (CHU9D) scores [6]. CHU9D scores will be transformed into individual health-related quality of life scores, which will be used to estimate QALYs over the trial period, using the area under the curve method. We will compare costs and health outcomes between the two intervention arms over time using generalized linear models.

To ascertain whether the intervention is cost-effective, we will calculate an incremental cost-effectiveness ratio, as the ratio between the difference in costs and the difference in QALYs or responders between interventions, for each costing perspective and express them as cost per additional QALY and cost per responder. We will explore uncertainty around the cost and outcome estimates and perform relevant sensitivity analyses.

6. **References**

1. Phillips KA, Hollander E, Rasmussen SA, Aronowitz BR, DeCaria C, Goodman WK. A severity rating scale for body dysmorphic disorder: development, reliability, and validity of a modified version of the Yale-Brown Obsessive Compulsive Scale. Psychopharmacology Bulletin. 1997;33:17-22.

2. Monzani B, Fallah D, Rautio D, Gumpert M, Jassi A, Fernández de la Cruz L, Mataix-Cols D, Krebs G. Psychometric Evaluation of the Yale-Brown Obsessive-Compulsive Scale Modified for Body Dysmorphic Disorder for Adolescents (BDD-YBOCS-A). Child Psychiatry & Human Development. 2023;54:1799-1806.

3. Hopewell S, Chan AW, Collins GS, Hrobjartsson A, Moher D, Schulz KF, Tunn R, Aggarwal R, Berkwits M, Berlin JA, Bhandari N, Butcher NJ, Campbell MK, Chidebe RCW, Elbourne D, Farmer A, Fergusson DA, Golub RM, Goodman SN, Hoffmann TC, Ioannidis JPA, Kahan BC, Knowles RL, Lamb SE, Lewis S, Loder E, Offringa M, Ravaud P, Richards DP, Rockhold FW, Schriger DL, Siegfried NL, Staniszewska S, Taylor RS, Thabane L, Torgerson D, Vohra S, White IR, Boutron I. CONSORT 2025 statement: updated guideline for reporting randomised trials. Lancet. 2025;S0140-6736(25)00672-5.

4. Husereau D, Drummond M, Augustovski F, de Bekker-Grob E, Briggs AH, Carswell C, Caulley L, Chaiyakunapruk N, Greenberg D, Loder E, Mauskopf J, Mullins CD, Petrou S, Pwu RF, Staniszewska S, Force CIGRPT. Consolidated Health Economic Evaluation Reporting Standards 2022 (CHEERS 2022) statement: updated reporting guidance for health economic evaluations. BMJ. 2022;376:e067975.

5. Bouwmans C, De Jong K, Timman R, Zijlstra-Vlasveld M, Van der Feltz-Cornelis C, Tan Swan S, Hakkaart-van Roijen L. Feasibility, reliability and validity of a questionnaire on healthcare consumption and productivity loss in patients with a psychiatric disorder (TiC-P). BMC Health Services Research. 2013;13:217.

6. Lindvall K, Vaezghasemi M, Feldman I, Ivarsson A, Stevens KJ, Petersen S. Feasibility, reliability and validity of the health-related quality of life instrument Child Health Utility 9D (CHU9D) among school-aged children and adolescents in Sweden. Health and Quality of Life Outcomes. 2021;19:193.
