# Supplementary material for: Efficacy and cost-effectiveness of Internet-delivered cognitive-behavior therapy for children and adolescents with body dysmorphic disorder: study protocol for a randomized controlled trial
Source: Trials. 2026 Aug 31;27:557. doi: 10.1186/s13063-026-09974-z (PMC13527960; doi:10.1186/s13063-026-09974-z)
Supplement: Supplementary file 5 [file 13063_2026_9974_MOESM5_ESM.docx]

Dnr 2023-02193-01

Linköping Ward 2 Medicine

# DECISIONS

2023-04-26

**Applicant research principal**

Karolinska Institutet

**Researchers carrying out the project**

Lorena Fernández de la Cruz

**Project title**

A randomized controlled trial on internet-mediated cognitive behavioral therapy for adolescents with dysmorphophobia.

**Application details**

The application was received by the Ethical Review Authority on 2023-03-31 and became valid on 2023-04-06.

The Ethical Review Authority decides as follows.

**DECISIONS**

The ethical review authority approves the research specified in the application.

**2023-05-08**

**2023-02193-01-404603**

**The Ethical Review Authority**

Dnr 2023-02193-01

Linköping Ward 2 Medicine

On behalf of the Ethical Review Authority

Anna Lander Chairman

The decision has been made by the following persons:

**Chairman**

Anna Lander (Alderman)

**Members with scientific expertise**

Cecilia Gunnarsson (Hereditary Cardiovascular Diseases, Genetics, Syndromes, Scientific Secretary)

Martin Hägglund (physiotherapy, epidemiology, scientific secretary)

Karin Åkesson (Paediatrics, rapporteur)

Erik Lexne (Psychiatry)

Preben Kirkegaard Kjölhede (Obstetrics/Gynecology. Gynecological Oncology.)

Ulrika Müssener (Public Health)

Camilla Nilsberth (Geriatrics, Neurobiology)

Håkan Olausson (Clinical Neurophysiology)

Per Sandström (Surgery)

**Members representing the public interest**

Martina Aronsson

Per-Arne Fall

Fredrik Hasselgren

Bertil Nilsson

Stefan Redéen

**The decision is sent to**

Principal investigator: Lorena Fernández de la Cruz

Principal of the research: Mats Olsson

**2023-05-08**

**2023-02193-01-404603**

**The Ethical Review Authority**
